# Novel compounds targeting the enterohemorrhagic *Escherichia coli* type three secretion system reveal insights into mechanisms of secretion inhibition

**DOI:** 10.1111/mmi.13719

**Published:** 2017-06-14

**Authors:** Riccardo Zambelloni, James P.R. Connolly, Alejandro Huerta Uribe, Karl Burgess, Rodolfo Marquez, Andrew J. Roe

**Affiliations:** ^1^ Institute of Infection, Immunity & Inflammation, College of Medical, Veterinary and Life Sciences University of Glasgow Glasgow G12 8QQ UK; ^2^ Department of Chemistry Xi'an Jiaotong‐Liverpool University SIP Suzhou 215123 China

## Abstract

Anti‐virulence (AV) compounds are a promising alternative to traditional antibiotics for fighting bacterial infections. The Type Three Secretion System (T3SS) is a well‐studied and attractive AV target, given that it is widespread in more than 25 species of Gram‐negative bacteria, including enterohemorrhagic *E. coli* (EHEC), and as it is essential for host colonization by many pathogens. In this work, we designed, synthesized and tested a new series of compounds that block the functionality of the T3SS of EHEC. Affinity chromatography experiments identified the primary target of the compounds as the T3SS needle pore protein EspD, which is essential for effector protein translocation into host cells. These data were supported by mechanistic studies that determined the coiled‐coil domain 1 of EspD as a key compound‐binding site, thereby preventing correct assembly of the T3SS complex on the cell surface. However, binding of inhibitors to EspD or deletion of EspD itself did not result in transcriptional down‐regulation of effector proteins. Instead, we found the compounds to exhibit dual‐functionality by also down‐regulating transcription of the entire chromosomal locus encoding the T3SS, further demonstrating their desirability and effectiveness.

## Introduction

Antibiotic resistance is increasing among common bacterial pathogens and is now considered a global threat by the World Health Organization (http://www.who.int/en/). Anti‐virulence (AV) therapies are a promising alternative to traditional antibiotics for fighting bacterial infections. A key feature of this strategy is that ‘virulence‐blocking’ mechanisms designed to target only the functionality of virulence factors carried by pathogens. This specificity helps avoid effects on the endogenous microflora and thereby exerts less selective pressure, reducing the development of resistance (Rasko and Sperandio, 2010; Beckham and Roe, 2014; Allen *et al*., 2014). One potential target for AV therapy is the bacterial type three secretion system (T3SS), a conserved protein injection organelle that is central to the virulence of over 25 species of human, animal, and plant pathogens including enteropathogenic and enterohemorrhagic *E. coli* (EHEC)*, Chlamydia spp, Pseudomonas aeruginosa, Salmonella spp., Shigella spp., Citrobacter rodentium* and *Yersinia spp*. (Cornelis, 2006; Keyser *et al*., 2008; Büttner, 2012; Collins *et al*., 2014). The T3SS is a complex needle‐like structure that penetrates the host cell membrane and functions as a conduit to translocate virulence effectors, intimately attach and ultimately creating a niche to allow the pathogen to survive (Cornelis, 2006; Büttner, 2012).

Colonization of both humans and animals by EHEC is highly dependent on its T3SS, encoded on a large horizontally acquired pathogenicity island, which enables intimate adhesion to and subversion of host epithelial intestinal cells. A key feature of this process is the development of attaching and effacing (A/E) lesions on the host cell membrane, defined by pedestal‐like cytoskeletal rearrangements induced by T3SS effector proteins (Moon *et al*., 1983; McDaniel *et al*., 1995; Nataro and Kaper, 1998; Frankel *et al*., 1998; Wong *et al*., 2011). The expression, regulation and function of the T3SS is complex and has been researched and reviewed extensively (Cornelis, 2006; Mellies *et al*., 2007; Büttner, 2012; Connolly *et al*., 2015). Three T3SS components most relevant to this study are EspA, EspD and Tir. The needle‐like structure that spans the bacterial membrane and allows effector proteins to be delivered into host cells is assembled by the polymerization of EspA (Knutton *et al*., 1998; Yip *et al*., 2005). The EspA needle is then capped by EspD, a 39 kDa protein predicted to comprise of two coiled‐coil domains for host cell membrane anchoring and homo‐oligomerization, two transmembrane domains for insertion and two amphipathic regions involved in chaperone recognition (Daniell *et al*., 2001; Dasanayake *et al*., 2011). EspD subunits insert into the host cell membrane creating a translocation‐pore allowing the injection of a suite of effector proteins into the host cell milieu (Shaw *et al*., 2001; Chatterjee *et al*., 2015). Arguably the most important of these effectors is Tir, which is integrated into the membrane of the host cell following its translocation into the host cell cytosol. Tir serves as a receptor for intimin (Translocated intimin receptor) and the interaction between these two components initiates reorganization of the host cell cytoskeleton and intimate attachment (Kenny *et al*., 1997).

A well studied class of AV compounds, the salicylidene acylhydrazides (SAs), were originally identified as putative T3SS inhibitors in *Yersinia pseudotuberculosis* (Kauppi *et al*., 2003; Nordfelth *et al*., 2005). The compounds were shown to be broadly effective in inhibiting the function of the T3SS in a number of pathogenic bacteria including *Chlamydia* spp., *Salmonella typhimurium, Shigella* spp. and EHEC (Muschiol *et al*., 2006; Bailey *et al*., 2007; Hudson *et al*., 2007; Tree *et al*., 2009; Veenendaal *et al*., 2009). To help understand the molecular mechanism by which the SAs affect the T3SS, we previously identified the cellular targets for this group of compounds and showed that their mode of action likely results from a synergistic effect arising from a perturbation of the function of several conserved metabolic proteins (Wang *et al*., 2011). We concluded that although effective, the SAs were rather promiscuous. The compounds also displayed poor solubility in biological buffers and proved to be prone to hydrolysis in acidic aqueous conditions. Therefore, the aim of our study was to try and develop novel compounds with better stability and selectivity. Accordingly, we have identified a new class of AV compounds capable of inhibiting the T3SS in a highly selective manner. We have demonstrated that the translocation pore protein EspD acts as the primary target for these compounds resulting in inhibition of effector secretion and that unlike their counterpart SA compounds, there is no detrimental effects on bacterial growth or viability thus fulfilling one of the key criteria of AV therapy. This comprehensive analysis provides a baseline for future work and development of this effective class of T3SS inhibitor compounds.

## Results

### Synthesis of new SA derivative compounds

**Figure 1:**
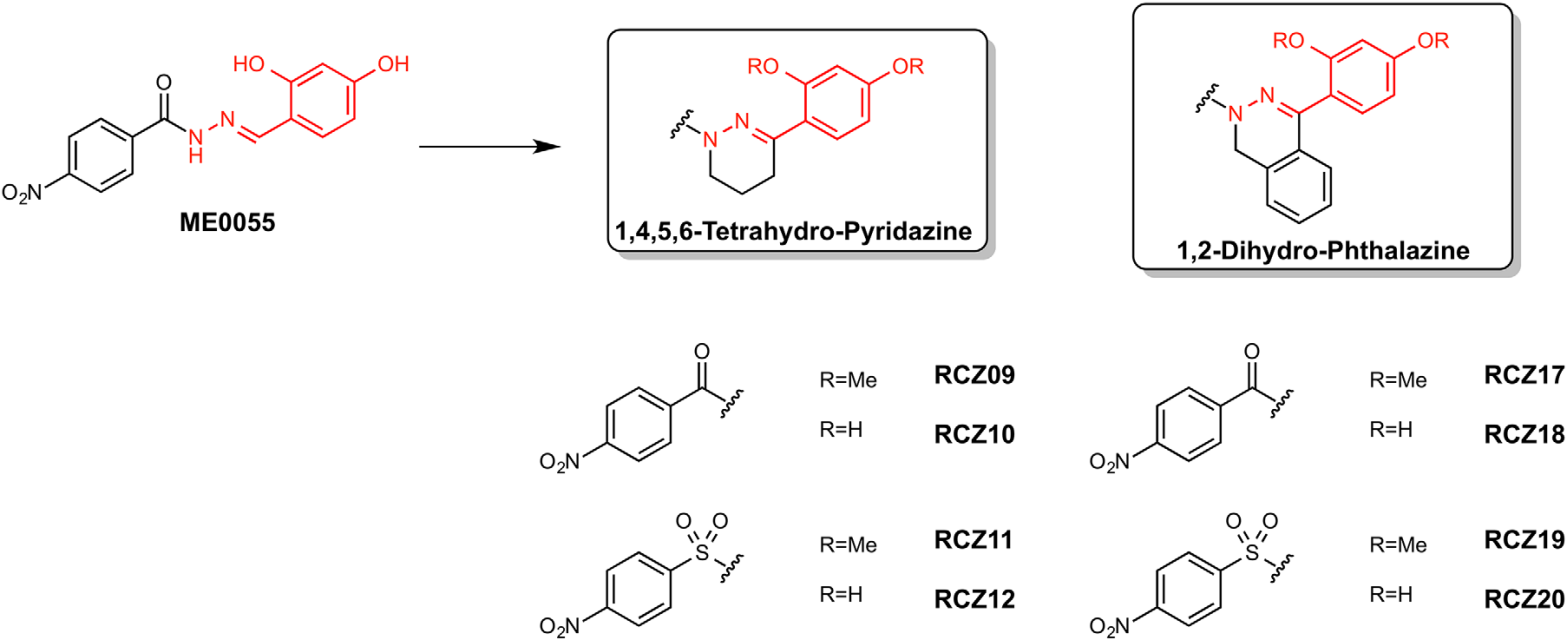
Overview of the series of compounds generated in this study. The imine motif of ME0055 was stabilized in two hydrazine‐containing heterocycles: 1,4,5,6‐tetrahydro‐pyridazine and 1,2‐dihydro‐phthalazine (THP and DHP). Around this core, both carbonyl and sulfonyl groups were incorporated to provide diversity. Similarly, methyl substitutions of the hydroxyl groups on the THP and DHP cores were generated.

We have previously demonstrated that the salicylidene acylhydrazide compound ME0055 is a potent inhibitor of the T3SS of EHEC (Tree *et al*., 2009; Wang *et al*., 2011). However, although effective, the compound was shown to be rather promiscuous, interacting with several bacterial proteins. Moreover, ME055 is poorly soluble in biological buffers and is prone to hydrolysis in acidic aqueous conditions. Therefore, it was decided to modify the ME0055 structure by incorporation of the imine moiety into two different hydrazine‐containing heterocycles, 1,4,5,6‐tetrahydropyridazine (THP) and 1,2‐dihydrophthalazine (DHP) (Fig. 1). Key substituents on both phenyl rings were retained for a direct comparison with ME0055. The synthesis of our derivatives (see Supporting Information Materials and Methods) started from resorcindimethylether (1), which underwent Friedel‐Crafts acylation with both succinic or phthalic anhydrides to afford carboxylic acids intermediates 2 and 3. Pyridazinone 4 and phthalazinone 5 were obtained in excellent yield by treating 2 and 3 with hydrazine hydrate in refluxing ethanol. Lithium aluminum hydride reduction afforded the corresponding amines 6 and 7. Acylation with 4‐nitrobenzoyl chloride subsequently achieved the di‐methoxy analogues RCZ09 (THP) and RCZ17 (DHP) in moderate yield. After trying different demethylation conditions, we obtained the di‐hydroxyl derivatives RCZ10 (THP) and RCZ18 (DHP) with boron tribromide at 40°C. A sulfonyl version of both the THP and DHP derivatives was also synthesized to expand the series. Amines 6 and 7, were consequently sulfonylated with nosyl chloride to achieve the corresponding dimethoxy‐sulfonyl analogues RCZ11 (THP) and RCZ19 (DHP). Demethylation was performed following the previous conditions to afford the sulfonyl di‐hydroxyl molecules RCZ12 (THP) and RCZ20 (DHP). Schematic synthesis maps can be seen in Supporting Information Fig. S1.

### Biological evaluation of the novel AV compounds

**Figure 2:**
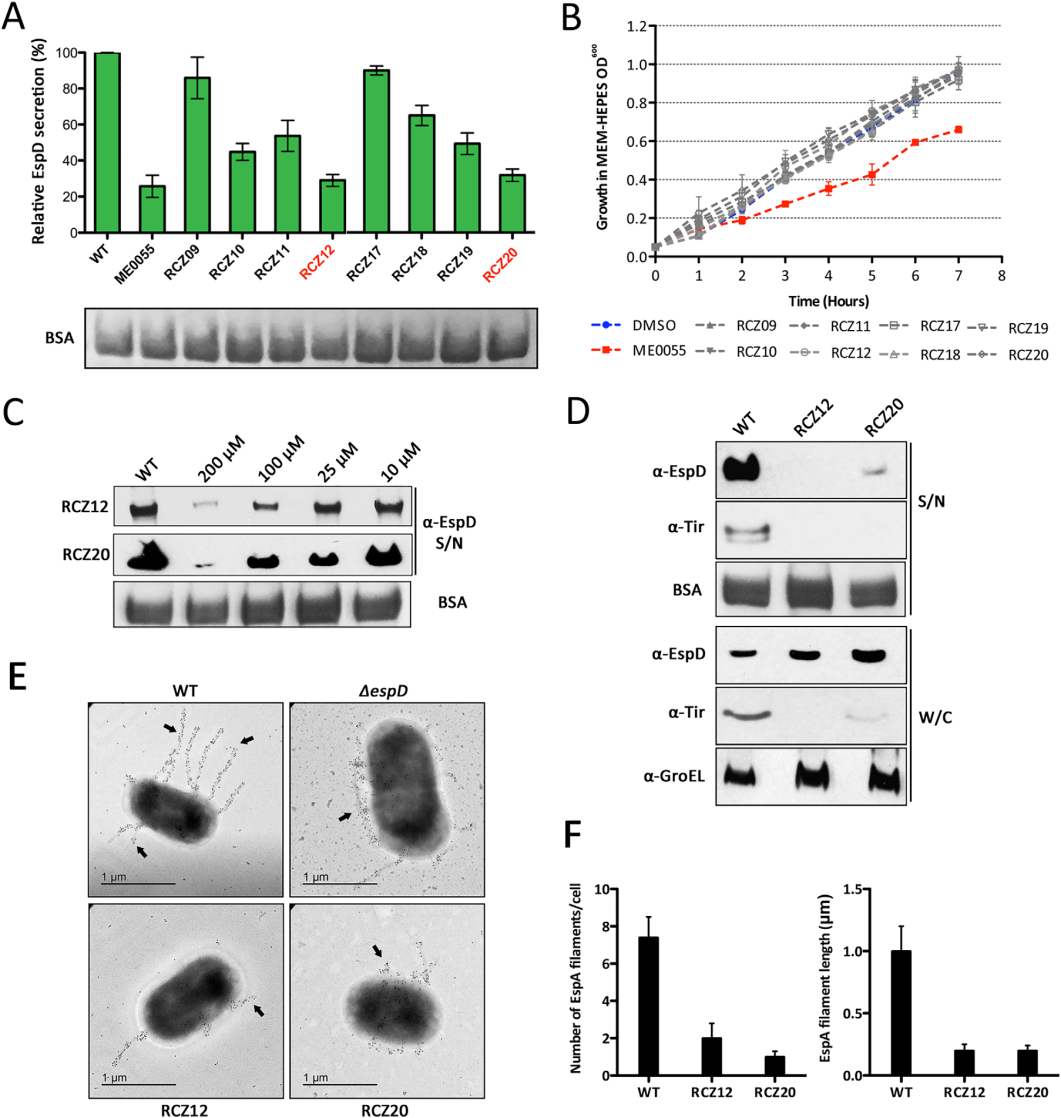
Biological evaluation of novel T3SS inhibitor compounds. A. Total secreted protein levels were assayed in cultures of EHEC grown in MEM‐HEPES with or without the series of novel compounds (100 μM) and probed for relative EspD levels by immunoblot. The data are represented as a percentage of total EspD secreted in the wild type (WT) background. ME0055 was used as a positive control for secretion inhibition. Samples were normalized according to the OD^600^ of the culture and the equal addition of exogenous BSA was used as a loading control. B. Growth curves of EHEC cultured in MEM‐HEPES supplemented with the novel derivatives described in panel A. DMSO was used as a control given its use as the compound solvent. C. Concentration dependent inhibition of type 3 secretion by RCZ12/20. Immunoblot analysis of secreted EspD levels after treatment of bacterial cultures with 10–200 μM of RCZ12/20. D. Comparative analysis of EspD and Tir secretion as well as expression in whole cell lysates from cell cultures treated with 200 μM of RCZ12/20. The secreted supernatant (S/N) and whole cell lysate (W/C) are labeled accordingly. Levels of GroEL and exogenous BSA were probed in the W/C and S/N fractions respectively to assess equal protein loading. E. Scanning electron microscopy (SEM) analysis of T3SS formation on WT cells and after treatment with RCZ12/20. Cultures were fixed for SEM and gold‐immunolabeling of EspA was performed to visualize T3SS filament formation (black arrows). The *ΔespD* mutant strain was used as a control. All experiments described were performed at least in biological triplicate. F. Enumeration of EspA filament number per cell and filament length (μm) of EHEC cells before and after treatment with RCZ12/20 as shown in panel E.

ME0055 has previously been evaluated for activity against the T3SS of EHEC (Tree *et al*., 2009; Wang *et al*., 2011). Eight new compounds were generated for biological testing against EHEC: the two carbonyl di‐hydroxyl THP and DHP compounds, RCZ10 and RCZ18, with their corresponding carbonyl di‐methoxy counterpart, RCZ09 and RCZ17, and the two sulfonyl di‐hydroxyl analogues, RCZ12 and RCZ20, with the sulfonyl di‐methoxy counterpart, RCZ11 and RCZ19 (Fig. 1). We screened the new compounds ability to block T3SS‐mediated protein secretion (using the secreted protein EspD as a probe), similarly to ME0055 (Fig. 2A). As expected, ME0055 showed a strong fivefold reduction of the protein secretion at 50 μM. Reduced secretion was also detected for our new candidates. Carbonyl di‐methoxy analogues (RCZ09 and RCZ17) did not reduce EspD secretion in a significant way, while the sulfonyl di‐methoxy molecules (RCZ11 and RCZ19) had a slightly stronger effect than the carbonyl counterpart, decreasing of twofold the relative secretion of the protein (Fig. 2A). The same pattern was detected for the four di‐hydroxyl compounds (RCZ10, 12, 18 and 20) with the sulfonyl di‐hydroxyl derivatives (RCZ12 and RCZ20) having a stronger inhibition profile than the carbonyl counterparts (RCZ10 and RCZ18). RCZ12 and RCZ20 displayed the most promising activity against EspD secretion, displaying a fourfold reduction in relative secretion (Fig. 2A). It was interesting to note that inhibition by the four di‐hydroxyl derivatives was generally more marked than the four di‐methoxy molecules, suggesting that the hydroxyl groups may be essential for activity. The effect exhibited by RCZ12 and RCZ20 was comparable to ME0055 activity and thus these two compounds were carried forward for further evaluation.

Since we were testing new potential AV compounds, which should target only virulence factors (Allen *et al*., 2014), the bacterial growth in presence of our new candidates was evaluated at a concentration of 100 μM (Fig. 2B). Optical density (OD_600_) of bacteria grown in T3SS‐inducing media (MEM‐HEPES) was measured hourly. ME0055 was demonstrated to have a measurable effect the growth rate at this concentration, as previously reported (Tree *et al*., 2009), whereas the new THP and DHP derivatives did not affect bacterial growth. Furthermore, we tested the viability of bacteria grown in high concentrations of our compounds by colony count analysis. Encouragingly, the RCZ compounds did not affect the bacterial viability even at 200 μM. Targeted metabolomic analysis of whole cell lysates and supernatants also confirmed the uptake of our compounds into the bacterial cytosol, eliminating any concerns about compound suitability. The signal intensity of RCZ12/20 from the bacterial cytosolic fraction was approximately 9.2‐ and 9.5‐fold (log^2^) higher than that detectable in the supernatant respectively (Supporting Information Fig. S2). These results provide further evidence for the suitability of our compounds as AV inhibitor candidates. A further desirable property of the compounds is better stability in acidic conditions. Incubation of ME055 in 20% HCl and 1H NMR analysis over a time course indicated hydrolysis within 2 h with the appearance of the aldehyde peak at 9.2 ppm corresponding to the 2,4‐dihydroybenzaldehyde and disappearance of the imine hydrogen at 8.7 ppm (Supporting Information Fig. S3A–C). In contrast RCZ12 showed no evidence of hydrolysis in the presence of acid over the 12 h time course tested (Supporting Information Fig. S3D and E).

We next began to explore the mode of action of the new compounds. First, we evaluated the efficacy of RCZ12 and RCZ20 across a range of concentrations from 10 to 200 μM (Fig. 2C). The repression of EspD secretion was concentration dependent, with measurable inhibition appearing at ∼25 μM and increasing with the concentration gradient, greater than fivefold at 200 μM. Second, we performed immunoblot analysis on secreted protein fractions for a second secreted protein, Tir. Similarly to EspD, Tir secretion was undetectable when grown in the presence of either RCZ12 or RCZ20 at 200 μM (Fig. 2D). To address whether these results indicate inhibition of protein secretion or an actual inhibition of Tir/EspD production we also probed the whole cell lysate with EspD and Tir antibodies. Intriguingly, EspD was found to accumulate inside the cells despite inhibition of secretion whereas Tir production was actually decreased in whole cell lysates (Fig. 2D). This is contrast to ME0055, which inhibits both production and secretion of EspD and thus suggests a distinct mode of action. Total protein loading levels were normalized by probing for the non‐virulence, heat shock protein, GroEL, which was unaffected by the compounds. These results indicated that our new derivatives likely act in a different manner to the SA compound. While ME0055 activity seemed to be blocking the T3SS apparatus at transcriptional level, both RCZ12 and RCZ20 mode of action seemed to be more focused on negatively affecting the ability of T3SS to translocate effector proteins such as EspD and Tir.

Immunoblot analyses suggested that blocking protein secretion rather than production may be mediating T3SS repression exhibited by our new compounds. Indeed, we see little affect on the intracellular production of EspD but protein secretion remains inhibited. We reasoned that this could be due to a post‐transcriptional mechanism. Indeed, expression of EspD is known to be essential for the formation of T3SS filament structures on the bacterial cell surface (Kresse *et al*., 1999). Therefore, we investigated this by way of scanning electron microscopy. Wild type EHEC produced an average of 8 long EspA needle formations (∼1000 nm) per bacterium, while a Δ*espD* mutant did not produce extended needle structures (Fig. 2E). RCZ12 and RCZ20 treated bacteria similarly had fewer characteristic T3SS needles on their cell surface (Fig. 2E) with an average of 1–2 needles per bacterium (Fig. 2F). These cells were comprised of shorter needles than that of the wild type, averaging in ∼200 nm in length (Fig. 2F). Furthermore, there was an apparent accumulation of EspA at the cell surface of RCZ12/20 treated bacteria, which suggests an aborted T3SS apparatus (Fig. 2E).

### Identification of RCZ12/20 cellular targets

Phenotypic evaluation of compounds RCZ12 and RCZ20 suggested inhibition of the T3SS by interference of needle assembly and protein secretion. To determine how this inhibition was taking place we performed whole cell lysate pull‐down experiments using biotinylated derivatives of RCZ12/20. We reasoned that since a reduction of EspD secretion was detected with the sulfonyl di‐methoxy analogues in our preliminary assays, the two hydroxyl groups on the right‐hand side of the molecule may be essential for the interaction. We therefore chose to insert the biotin tag on the left‐hand side of our candidates.

The synthesis of biotinylated RCZ12/20 (Supporting Information Materials and Methods) began as treatment with allyl bromide to generate intermediates 8 and 9 in excellent yields. Nitro reduction was carried out in mild conditions in the presence of iron and an aqueous solution of NH_4_Cl in refluxing ethanol. Intermediates 10 and 11 underwent acylation with hex‐5‐ynoyl chloride to obtain both alkyne intermediates 12 and 13. Huisgen copper catalyzed 1,3‐dipolar cycloaddition between the alkyne intermediates, 12 and 13, and biotin‐N3 was performed in presence of copper(II)sulfate and sodium ascorbate in a 3:1 mixture of THF and water at 50°C (31). The protected biotin‐labeled RCZ12 and RCZ20 products 14 and 15 were de‐protected with tetrakis(triphenylphosphine)palladium in refluxing methanol (32), thus resulting in biotin‐RCZ12 and biotin‐RCZ20 synthesized in 5 steps. Schematic synthesis maps can be seen in Supporting Information Fig. S4.

**Figure 3:**
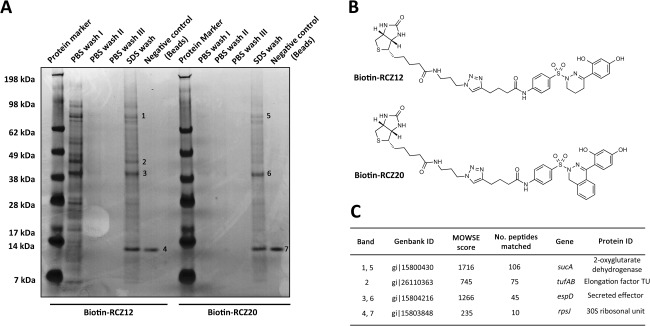
Biotin‐Streptavidin affinity pulldown assay of RCZ12/20 with whole cell lysate of EHEC. A. Coomassie stained SDS‐PAGE gel of biotin‐RCZ12/20 bound proteins. Each wash and elution stage is indicated above each well. The negative control for nonspecific binding corresponds to the assay performed using Streptavidin beads alone. The experiment was performed in triplicate. B. The chemical structure of the biotin labeled RCZ12 and RCZ20 compounds used in the pull‐down assays. C. Table of results highlighting the targets of RCZ12/20 as identified by tandem mass spectrometry. The band number, genBank/protein ID, MOWSE score and gene name are indicated.

Streptavidin‐coated magnetic beads were utilized to perform the pull‐down assay with our two biotinylated compounds, due to the high affinity between biotin and streptavidin. The beads were firstly incubated with the biotin derivatives and then with EHEC whole cell lysate from cells cultured in MEM‐HEPES. The beads were washed to remove nonspecifically bound protein and boiled in SDS to denature the bound protein. Boiled protein samples bound to biotinylated RCZ12/20 were separated and visualized by SDS‐PAGE followed by tandem mass spectrometry analysis for identification (Fig. 3A). The magnetic beads were also incubated with the whole cell lysate from cells cultured without biotinylated RCZ12/20 as a control for nonspecific interactions (Fig. 3A). Biotin‐RCZ12 and biotin‐RCZ20 (Fig. 3B) were found to have an almost identical banding pattern indicating affinity for similar proteins and likely the same cellular targets (Fig. 3C). Interestingly, the most abundant band identified in the assay corresponded to EspD (MOWSE score/# peptides matched: 1266/45 for RCZ12 and 1174/40 for RCZ20), along with 2‐oxoglutarate dehydrogenase (1716/106) and elongation factor Tu (Ef‐Tu) (745/75) (Fig. 3C). Both the 2‐oxoglutarate‐dehydrogenase and Ef‐Tu were considered as ‘off‐target’ proteins as they are not directly involved in the EspD secretion process or T3SS assembly. An additional band identified corresponded to 30S ribosomal subunit S10 (235/10) but this was demonstrated to be a nonspecific interaction bound to streptavidin rather than our biotinylated compounds, as indicated in the negative control samples. Thus EspD appeared to be the only genuine T3SS‐related target that showed affinity for both our biotinylated compounds. These results suggest that the cellular target of RCZ12/20 is the translocation pore protein EspD and that this interaction could explain the inhibition of protein secretion and EspA needle formation without any effects on EspD production. Furthermore, the target identification of our new compounds shows a drastic improvement in selectivity over the previous SA compound ME0055, which reacted with 14 cellular targets in a pull‐down assay, none of which were T3SS related proteins (Wang *et al*., 2011).

### Analysis of EspD as a cellular target of the T3SS inhibitors RCZ12/12

**Figure 4:**
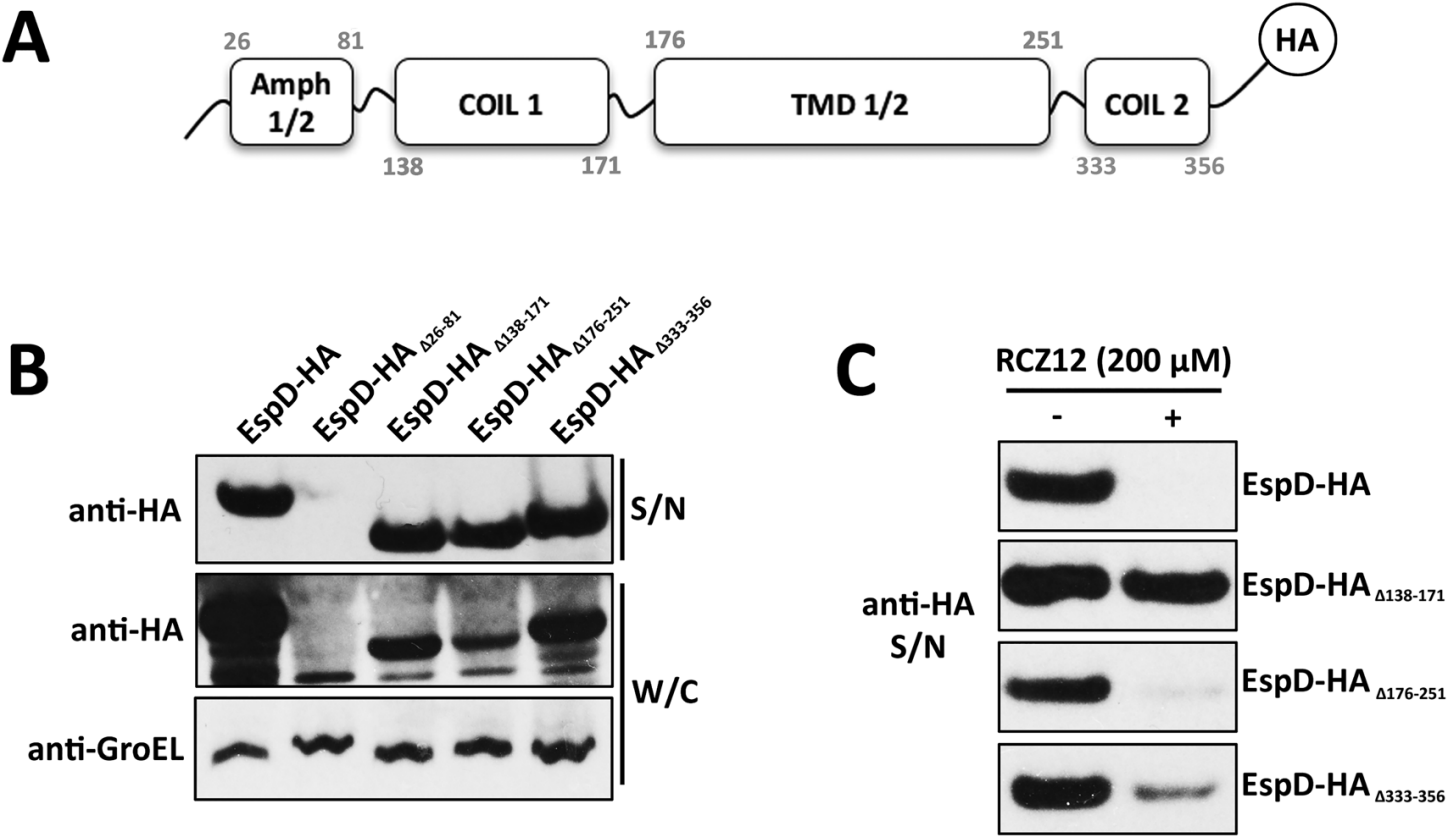
Identification of AV compound binding sites to EspD. A. Schematic representation of the HA‐tagged EspD peptide sequence from EHEC highlighting the relevant functional domains – the amphipathic 1 and 2 regions (amino acids 26–81), the coiled‐coil domain 1 (amino acids 138–171), the transmembrane domains 1 and 2 (amino acids 176–251), the coiled‐coil domain 2 (amino acids 333–356) and the HA tag. A full length EspD‐HA derivative was generated as well as HA‐tagged truncations in each of the above functional domains. B. Immunoblot analysis of EspD‐HA derivative expression and secretion. EHEC whole cell lysates (W/C) and secreted protein from supernatants (S/N) were probed with anti‐HA antibody. The shift in molecular weight due to truncations can be seen clearly when compared to the full length EspD‐HA. Anti‐GroEL levels were probed from the W/C fraction to normalize protein loading. C. Identification of an RCZ12 EspD inhibition site. Immunoblot analysis of EspD‐HA levels in S/N fractions from EHEC cells cultured with (+) and without (–) 200 μM RCZ12 in MEM‐HEPES. The effects of RCZ12 treatment on EspD‐HA derivative secretion were compared to the full length EspD‐HA, which was inhibited fully in a similar manner to WT EspD. Immunoblot experiments were all performed in triplicate.

Currently no structural data exists for EspD making predictions as to the likely binding site problematic. To overcome this, we used a genetic approach to mutate functional domains of EspD (Fig. 4A) and investigate activity with RCZ12/20. Mutant strains of EHEC containing truncated EspD coding regions, each tagged with the HA epitope to allow probing by western blot, were generated by allelic exchange. A total of 5 mutants were generated. First, we created a full‐length EspD‐HA strain. Next, we sequentially deleted 4 essential regions of the protein coding sequence – the 2 amphipathic regions (EspD‐HA_Δ26–81_), the coiled‐coil domain 1 (EspD‐HA_Δ138–171_), the 2 transmembrane domains (EspD‐HA_Δ176–251_) and the coiled‐coil domain 2 (EspD‐HA_Δ333–356_). We assessed the ability of our mutant strains to express and secrete the modified EspD‐HA derivatives. The mutant strains were cultured in MEM‐HEPES and both the supernatant and whole cell lysate were analyzed by western blot (Fig. 4B). All EspD‐HA derivatives except for EspD‐HA_Δ26–81_ were detectable in both cellular fractions. As previously observed, deletion of the 2 amphipathic regions generated an unstable construct (EspD‐HA_Δ26–81_), which cannot be recognized by its chaperone and is rapidly degraded (Dasanayake *et al.*, 2011). These data demonstrate that four of our mutant strains can produce detectable EspD truncates that are capable of being secreted.

Next, we assessed the ability of our compound RCZ12 to inhibit protein secretion of the truncated EspD mutant forms. The C‐terminus tagged EspD‐HA did not result in any reduced inhibition as RCZ12 totally inhibited EspD‐HA secretion at 200 μM, as seen in the WT protein (Fig. 4C). Conversely, a complete loss of activity was detected for EspD‐HA_Δ138–171_, while retention of activity was observed for both EspD‐HA_Δ176–251_ and EspD‐HA_Δ333–356_ (Fig. 4C). These results strongly support the notion that the EspD coiled‐coil domain 1 acts as an interacting partner of RCZ12/20, thus confirming our hypothesis of an inhibitory interaction between our compounds and EspD. We can therefore postulate that RCZ12/20 accumulate in *E. coli* and subsequently bind to intracellular EspD, inducing a conformational change that blocks the translocation of this protein and inhibits T3SS pore formation.

### Transcriptional effects induced by RCZ12/20 treatment of EHEC

The data presented suggests that our newly designed T3SS inhibitors RCZ12/20 function by binding EspD inside the bacterial cell, interfering with functionality of the translocation pore and thus inhibiting effector protein secretion. However, studies thus far have focused on RCZ12/20 binding to EspD and have not addressed the effects of these compounds on the transcription of virulence‐associated genes. Despite an accumulation of cellular EspD after compound treatment we observed decreased expression of Tir. This can be explained by one of two scenarios – either physical interference with EspD causes a negative transcriptional feedback to T3SS encoding genes or the compounds themselves also act at the level of transcription regulation. The latter was a characteristic of the original SA compounds and thus was also investigated (Tree *et al*., 2009).

**Figure 5:**
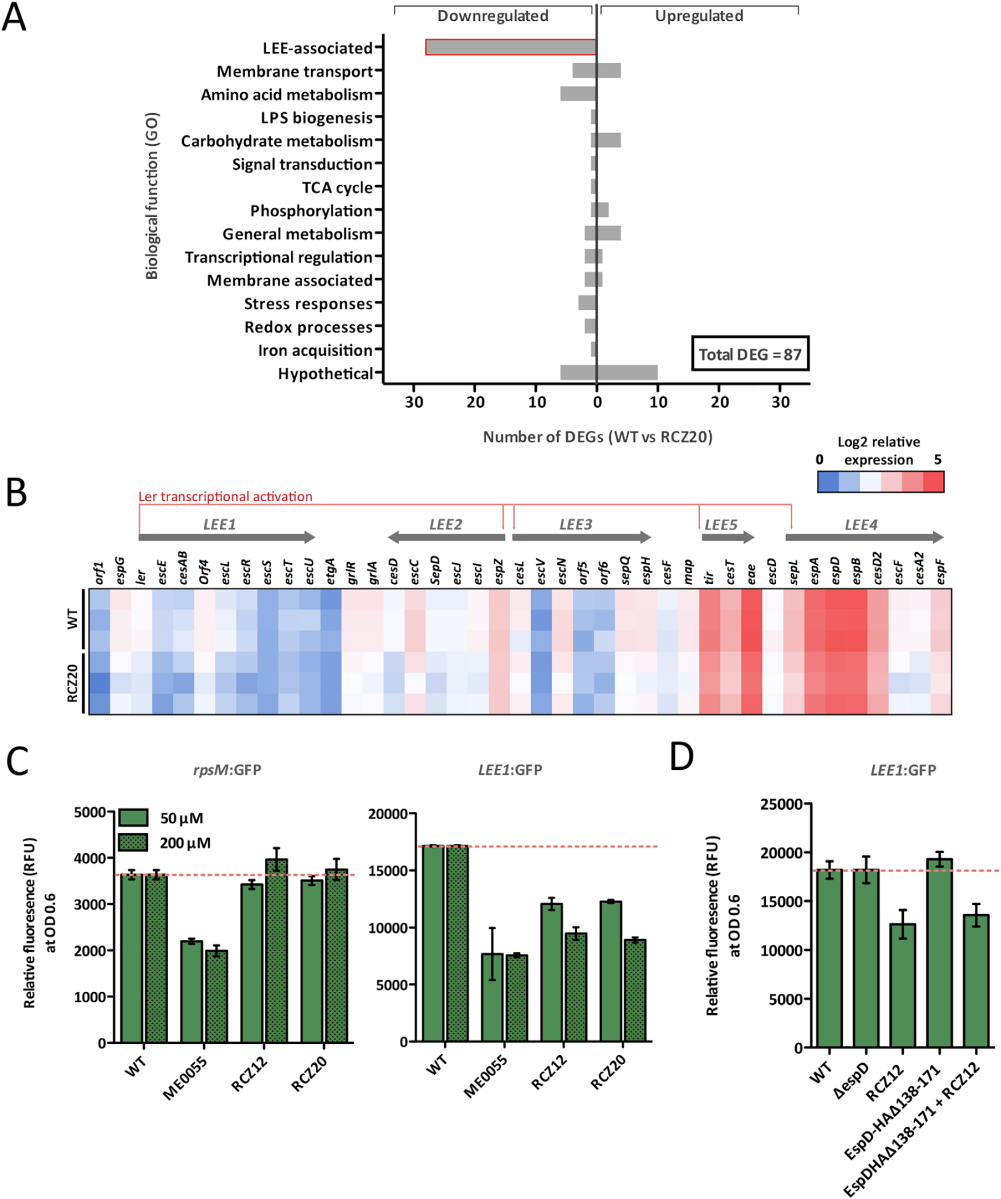
Characterization of the affects of RCZ20 treatment on transcriptional regulation of type 3 secretion in EHEC. A. RNA‐seq results of EHEC cultured in MEM‐HEPES with and without RCZ20. A gene was determined as differentially expressed if it displayed significant upregulation or downregulation with an FDR corrected *p* value of < 0.05. Three independent biological replicates were used in RNA‐seq experiments. B. Expanded view of the LEE island transcriptional landscape in response to RCZ20 treatment. The heatmap indicates the expression pattern (log^2^ normalized reads mapped) of each ORF within the LEE for each replicate. A schematic map of the LEE operon structure as well as master regulation by Ler is indicated above. C. Validation of RNA‐seq data using promoter‐GFP reporter fusions. Cell cultures carrying reporter fusion plasmids p*rpsM*:GFP (housekeeping gene expression) and p*LEE1*:GFP (LEE expression) were assayed under identical conditions to those performed in the RNA‐seq experiment for treatment with both RCZ12 and RCZ20 (50 and 200 μM). Promoter expression was measured as relative fluorescence units (RFU) and were normalized to OD^600^ 0.6. The expression level of either *rpsM* or *LEE1* in the WT background is indicated by the red dotted line. As a comparator, p*rpsM*:GFP and p*LEE1*:GFP activity were also measure in response to ME0055. D. RCZ12/20 compound activity exhibits dual functionality. p*LEE1*:GFP activity was measured in both the EspD‐HA and EspD‐HA_Δ138–171_ genetic backgrounds to investigate if the transcriptional effects exhibited by compound treatment are due to feedback from EspD inhibition. GFP reporter assays were performed in biological triplicate and are displayed as mean and standard error. E. Summary of RCZ12/20 dual‐functionality as AV compounds – primarily inhibition of EspD function by direct binding and mild transcriptional downregulation of the LEE with minimal off‐target effects.

We investigated these hypotheses by applying RNA‐sequencing (RNA‐seq) analysis to mRNA samples purified from cultures of EHEC grown in MEM‐HEPES with or without RCZ20. This gave us a ‘global’ high‐resolution view of the bacterial transcriptomes under T3SS inducing conditions and in response to the novel inhibitors. Figure 5A shows that RCZ20 treatment resulted in differential expression of only 87 genes (FDR *p* < 0.05; Supporting Information Table S4). All the genes necessary for the formation of a fully functional T3SS are encoded on a 5 operon, polycistronic pathogenicity island named the locus of enterocyte effacement (LEE), which contains 41 open reading frames (ORFs) (McDaniel *et al*., 1995; Elliott *et al*., 1998). Strikingly, all 41 ORFs of the LEE were significantly down‐regulated after RCZ20 treatment, suggesting the compounds have some effect on global regulation of this pathogenicity island (Fig. 5B). Indeed, the ‘master regulators’ of the LEE, Ler and GrlRA, that control transcription of all 5 LEE operons are encoded within the island itself (Mellies *et al*., 1999; Sánchez‐SanMartín *et al*., 2001; Haack *et al*., 2003; Deng *et al*., 2004). Three other transcriptional regulators were differentially expressed in the data set – the phosphorelay response regulator *rcsA*, the acid response regulator *yhiW* and the biofilm regulator *bssR*. The Rcs phosphorelay and GAD acid response systems have previously been linked to LEE regulation but YhiW and RcsA were not found to be involved (Tatsuno *et al*., 2003; Tobe *et al*., 2005; Tree *et al*., 2011). *rcsA* is, however, known to be activated by Ler, therefore explaining downregulation of this gene (Bingle *et al*., 2014). In addition to transcriptional regulation and virulence there were numerous genes affected by RCZ20. However, functional classification of these genes failed to identify any particularly enriched roles of the affected genes. These included genes involved in processes such as general metabolism, membrane structure and transport or stress responses but the number of genes affected were low in each category suggesting either indirect or off target effects. Interestingly, the most enriched gene categories outside of the LEE were hypothetical genes (13 genes differentially expressed). Taken together these results indicate that RCZ20 treatment indeed results in transcriptional downregulation of the T3SS as well as differential regulation of other genes. However, the focus of the genes affected toward the LEE further the highlights the powerful selectivity of our compounds.

### RCZ12/20 exhibit dual functionality at the transcriptional and post‐transcriptional levels

The results of the RNA‐seq and EspD‐HA inhibition experiments seemed somewhat paradoxical. The result suggest two likely scenarios that can explain the findings – either physical inhibition RCZ12/20 with EspD leads to negative transcriptional feedback to the LEE or RCZ12/20 exhibit dual‐functionality as inhibitors. Deng *et al*. previously analyzed the effects of systematically deleting every ORF within the LEE of the murine pathogen *Citrobacter rodentium* (Deng *et al*., 2004). The study found that deletion of *espD* had no apparent feedback on regulation of the LEE or protein secretion, despite its essential role in type 3 secretion. To verify that deletion of *espD* does not affect LEE regulation in an EHEC background we constructed a *ΔespD* mutant strain of TUV 93–0 and employed a set of previously developed GFP‐reporters plasmids (p*rpsM*:GFP and p*LEE1*:GFP) (Roe *et al*., 2003). These comprise the promoters for either the small ribosomal protein (RpsM) or the master regulator of the LEE (Ler) fused to GFP. Analysis of p*rpsM*:GFP activity in response to 50 and 200 μM RCZ12/20 and ME0055 confirmed that our compounds have no apparent detrimental effects on housekeeping gene expression unlike the previous SA compound, ME055 (Fig. 5C). Furthermore, analysis of p*LEE1*:GFP activity demonstrated that both RCZ12/20 and ME0055 treatment resulted in significant down‐regulation of the *LEE1* promoter (Fig. 5C). These results both confirm effectiveness of RCZ12/20 as T3SS inhibitors and also validate the RNA‐seq results described above.

To address the question of EspD feedback on the LEE we analyzed p*LEE1*:GFP activity in a *ΔespD* background. *LEE1* transcription was unaffected by deletion of *espD* (Fig. 5D). Strikingly, addition of RCZ12 to the *ΔespD* strain resulted in a significant decrease in p*LEE1*:GFP activity (Fig. 5D) indicating that irrespective of the absence of the target EspD, RCZ12 is still capable of transcriptional repression of the LEE, supporting the notion of RCZ12/20 dual‐functionality. The EspD‐HA inhibition experiments identified the EspD coiled‐coil domain 1 as the likely target of RCZ12/20 binding to this protein. To confirm that deletion of EspD did not result in artifactual results we performed the p*LEE1*:GFP activity analyses in an EspD‐HA_Δ138–171_ background. The results showed that modification of the coiled‐coil domain 1 did not affect *LEE1* transcription, however, treatment of this strain with RCZ12 indeed resulted in significant *LEE1* down‐regulation (Fig. 5D).

## Discussion

Resistance of bacterial pathogens to common antibiotics in considered a global threat and the majority of recent advances in antibiotic discovery have focused on Gram‐positive targeting drugs (Ling *et al*., 2015; Zipperer *et al*., 2016). AV therapy offers a promising alternative to more traditional antibiotic treatment (Rasko and Sperandio, 2010; Allen *et al*., 2014). Such an approach, however, requires a detailed understanding of the mechanisms behind an AV route to increase the focus on targeting virulence mechanisms specifically. Given its widespread nature and essential role in the pathogenesis but not survival of many Gram‐negative pathogens, the T3SS offers an attractive target for the development of novel AV therapies (Keyser *et al*., 2008).

Colonization of both humans and cattle by EHEC is highly dependent on the T3SS, encoded on the LEE pathogenicity island. Therefore, compounds that interfere with the expression and function of the T3SS offer an attractive proposition to combat this disease. Previous work had demonstrated that the SA class of compounds was capable of reducing expression of the T3SS. However, studies into their mechanism of action revealed multiple target proteins, many of which were associated with bacterial metabolic processes. Studies have used statistical molecular design to expand the library of SA class inhibitors (Dahlgren *et al*., 2010). In this work we wanted to replace the imine core with by the introduction of hydrazine‐containing heterocycles that would offer greater stability. Two different hydrazine‐containing rings were selected: the 1,4,5,6‐tetrahydro pyridazine (THP) and the 1,2‐dihydrophthalazine (DHP). The THP core includes two nitrogens in a six‐membered ring with a half‐chair conformation. In contrast, the DHP core has a completely planar conformation and the ortho‐fused phenyl ring gives a much greater hindrance in the central core of the molecule than the THP scaffold, also increasing the lipophilicity of the compound. To further expand the series, it was decided to replace the carbonyl function with a sulfone group, creating further derivatives for testing.

One key advantage of AV compounds compared with traditional antibiotics is the reduced selective pressure for the development of resistance. To this end, we demonstrate here that the new series of compounds do not inhibit the growth or viability of EHEC even at 200 μM, the highest concentration tested. However, only two of the new series were active at reducing secretion of known LEE encoded effector proteins. Both RCZ12 and RCZ20 resulted in the accumulation of EspD in the bacterial cytoplasm suggesting they might act by interfering with the normal secretion of this protein. Biotinylated derivatives of RCZ12/20 were used to help identify potential binding partners. Remarkably, we reproducibly identified EspD as a potential target of both lead compounds. While it would be desirable to show a direct, physical, interaction of RCZ12/20 with EspD this has proven problematic. EspD is produced in the bacterial cytoplasm and interacts with at least two chaperones, CesD and CesD2. Both appear important for the stability and secretion competence of EspD. Chatterjee *et al.* successfully purified recombinant EspD (Chatterjee *et al*., 2015) and kindly donated samples for testing but high concentrations of urea (8 M) and detergents had to be used to dissolve the protein making subsequent biophysical studies difficult to perform. For this reason we used a genetic approach to demonstrate that removal of the EspD coiled‐coil domain 1, previously shown to be important for T3SS mediated host cell binding, completely reverses the inhibitory effects of RCZ12, strongly supporting the notion that this is the site of compound binding to EspD. Physical interference with the translocation process can explain how we observe both an inhibition of secretion and cytoplasmic accumulation of EspD.

Intriguingly, our data suggest that RCZ12/20 do not work solely through binding to EspD. It was observed that while EspD accumulates intracellularly after RCZ12/20 treatment, this is not the case for Tir, which was both blocked for secretion and down‐regulated in its transcription. These data suggest some feedback to other members of the LEE through EspD blocking. The transcriptional down‐regulation observed by RNA‐seq showed that all 41 ORFs of the LEE were significantly repressed after RCZ20 treatment. Indeed, previous studies on the SA class of inhibitor compounds have demonstrated transcriptional down‐regulation of virulence factors, including the LEE T3SS. Further analysis of this associated transcriptional regulation using GFP‐reporters revealed that deletion of *espD* did not affect *LEE1* transcription despite its essential role in T3SS function and A/E lesion formation. Treatment with RCZ12, however, resulted in significant *LEE1* down‐regulation, similarly to RCZ20 by RNA‐seq. This suggests a dual functionality of these inhibitors: physical binding to EspD and disrupting its efficient translocation through the T3SS and transcriptional down‐regulation of the LEE by an undefined mechanism. However, we maintain that the primary and most effective mechanism of T3SS inhibition by RCZ12/20 is EspD interaction. This is supported by the finding that EspD‐HA_Δ138–171_ is still capable of being secreted after treatment with RCZ12/20 and despite mild down‐regulation of LEE transcription. Hence, the down‐regulation of the LEE by RCZ12/20 is not sufficient to explain the major phenotype, a distinct lack of effector protein secretion. Given the essential nature of translocation pore formation and establishment of host cell binding, along with the known hierarchy of T3SS effector translocation, this strategy of AV therapy is a viable option for therapeutic treatment of EHEC infections.

Given that the T3SS is central to the pathogenesis of a wide range of Gram negative bacteria, it would be interesting to test any effects of RCZ12/20 on related organisms. Both enteropathogenic *E. coli* (EPEC) and *C. rodentium* carry direct homologs of EspD. Interestingly, comparison of the EspD proteins from EHEC (EHEC str. Sakai), EPEC (O126:H6 str. E2348/69) and *C. rodentium* (str. ICC168) reveals several amino acid substitutions within the coiled‐coiled domain we propose to be a key target of RCZ12. The pathogens *Salmonella typhimurium*, *Shigella dysenteriae* and *Yersinia Pesudotuberculosis* also use pore‐forming effectors with similarity to EspD. However, despite sharing a conserved function, they are quite distinct to EspD with less than 27% protein identity. Therefore, extensive additional testing would be required to examine if RCZ12/20 are capable of affecting these other pathogens. Taken together, our study provides a comprehensive analysis of a new class of effective and highly selective T3SS inhibitors that should provide a baseline for future research and improvements.

## Experimental procedures

### Bacterial strains, plasmids and growth conditions

Strains and plasmids used in this study are listed in Supporting Information Tables S1 and S2 respectively. Generation of single gene deletion strains was carried out using the Lambda Red mediated recombination method, as described by Datsenko and Wanner (Datsenko and Wanner, 2000). Allelic exchange plasmids for generation of the truncated‐EspD‐HA strains (see below) were made using the NEBuilder HiFi DNA Assembly Cloning Kit (New England Biolabs) according to the manufacturer specifications and all the clones were confirmed by sequencing. Oligonucleotide primers (Invitrogen) are listed in Supporting Information Table S3. Bacterial strains were isolated from pure culture agar plates and used to inoculate liquid media. 5 ml overnight cultures were diluted to an optical density (OD^600^) of ∼0.05 in LB, M9 or MEM‐HEPES for induction of type 3 secretion (Sigma® M7278) supplemented with standard concentrations of antibiotics when required.

### Synthesis of chemical compounds

The synthesis, modification and purification of the compounds used in this study are detailed in the Supporting Information Materials and Methods. Compounds were dissolved in DMSO with the final DMSO concentration kept below 2% for all assays.

### Compound stability analyses

The stability of the compounds in acidic conditions was assessed using 1H NMR. Briefly, 15 mg of compounds were dissolved in DMSO‐d6 containing 20% hydrochloric acid (DCl) and DMSO‐d6 alone. 1H‐NMR spectra were recorded at 500 MHz using a Bruker Avancell 500 instrument. Samples were analyzed at *t* = 0 h, 2 h and 12 h after incubation.

### Analysis of compound uptake by targeted metabolomic profiling

Bacteria were grown in M9 minimal medium to an optical density of 0.9 and incubated with RCZ12 or RCZ20 for 60 min. Fractions were separated by centrifugation (2 min at 15 000 g). Bacterial pellets and supernatants were independently treated with a 1:1:3 chloroform/water/methanol solution and the resulting supernatants were analyzed by hydrophilic interaction liquid chromatography (HILIC) to measure their relative concentrations in each respective background. HILIC analysis was performed at the University of Glasgow Polyomics facility. Briefly, compounds were separated using a 15 cm × 4.6 mm ZIC‐pHILIC column (Merck Sequant) running at 300 uL/min. Mobile phase was A: 100% H_2_O with 20 mM ammonium carbonate and B: 100% Acetonitrile. Separation was performed on an UltiMate 3000 RSLC (Thermo) with a gradient running from 20% A, 80% B to 80% A, 20% B in 15 min, followed by a wash at 5% B for 3 min and equilibration at 20%A for 6 min for a total runtime of 24 min. Detection was performed using a Q‐Exactive (Thermo) at 70 000 resolution in positive/negative switching mode.

### Generation of HA‐tagged EspD and EspD truncates

Engineered EspD‐HA mutant strains were generated using allelic exchange, optimized for EHEC according to the protocol by Emmerson *et al*. (Emmerson *et al*., 2006). The method relies on the creation of temperature sensitive exchange plasmids containing homologous flanking regions that mediate insertion of the gene of interest through homologous recombination into the bacterial chromosome at their original location. Exchange plasmids for this process comprised of pIB307‐LEE4‐SacB/Kan (to generate the intermediate strain with the SacB/Kan cassette on the chromosome in place of *espD*) and pIB307‐LEE4‐EspD‐HA (to allow the incorporation of the HA epitope onto the coding frame of EspD in replacement of the SacB/Kan cassette). The plasmids only replicate at the permissible temperature of 28°C and are selectable on chloramphenicol. The initial recombination of SacB/Kan occurs at 42°C and is selectable on Kanamycin. The second and final recombination of the modified EspD epitope is negatively selected for with sucrose. Subsequently, pIB307‐LEE4‐EspD‐HA_Δ26–81_, pIB307‐LEE4‐EspD‐HA_Δ138–171_, pIB307‐LEE4‐EspD‐HA_Δ176–251_ and pIB307‐LEE4‐EspD‐HA_Δ333–356_ were created to allow the generation of 5 chromosomally encoded EspD‐HA truncates, each lacking the series of amino acids specified in the name of the plasmid.

### Affinity chromatography using biotin‐labeled RCZ12 and RCZ20

The pull‐down assay was performed using streptavidin‐coated magnetic beads (Dynabeads™ M‐280 Streptavidin from Invitrogen®). EHEC strain TUV93–0 was cultured at 37°C to an OD^600^ of ∼0.9. The cells were pelleted by centrifugation, washed several times with PBS and concentrated in PBS to a final volume of 2 ml. The suspended cells were lysed by sonication and kept under ice. The magnetic beads were aliquoted and washed with PBS using a magnetic rack. A 250 μM solution of Biotin‐RCZs in PBS:DMSO (10:1) was added to the beads and incubated shaking for 1 h at room temperature. The Biotin‐compound solution was removed and the beads were washed three times with PBS. 500 μl of the suspended cell lysate solution was then added to the tubes and incubated with shaking for another hour at room temperature. The lysate solution was removed and the beads were washed three times with PBS. Once washed, 15 μl of SDS sample buffer (Invitrogen, UK) was added to the tube and the beads were boiled at 95°C for 10 min to promote specific bound protein denaturation. Equals volumes of PBS washes and SDS samples were loaded onto a 4–12% Bis‐Tris gel, the proteins were separated through SDS‐PAGE electrophoresis and visualized by Coomassie Blue staining (Novex SDS‐PAGE system, Invitrogen). Specific bound proteins were excised for subsequent in‐gel digestion and analysis by tandem mass‐spectrometry. Proteins analyzed by peptide mass fingerprinting were given a MOWSE score to indicate the probability of the identification being correct. We set a threshold MOWSE value of 100 or greater, ensuring a significance of *p* < 10^−8^.

### Analysis of total secreted protein

Strains were cultured in prewarmed MEM‐HEPES with the desired compound concentration. Cultures were grown at 37°C to an OD^600^ ∼0.7 and then the solution was centrifuged for 10 min at 3750 rpm and 4°C to separate the cells from the supernatant. The cell pellets were lysed using BugBuster Protein Extraction buffer (Merck, New Jersey, USA), while the supernatant solution was treated with 10% (v/v) TCA at 4°C overnight. Secreted proteins were then centrifuged for 1 h at 3750 rpm, 4°C and re‐suspended in Tris‐HCl solution (pH 8.0). An equal amount of bovine serum albumin (BSA) was added to each secreted protein sample fraction to confirm equal loading of total secreted protein. Proteins from both whole cell and secreted fractions were analyzed by Western blot using the Novex system (Invitrogen) with primary antibodies EspD (1/5000), Tir (1/1000) or GroEL (1/10 000) and protein levels were compared by densitometry using ImageJ. Experiments were performed a minimum of three times to confirm the results.

### *In vitro* GFP reporter fusion assays

*In vitro* transcription assays were carried out using a series of reporter plasmids containing the promoter region of interest fusion in‐frame to eGFP, which were previously generated (Roe *et al*., 2003). Transformed strains were cultured in MEM‐HEPES at 37°C with shaking at 200 rpm. Hourly samples were extracted for measurement of optical density (growth) and fluorescence (relative promoter activity). GFP expression was measured in a black 96‐well plate (excitation at 485 nm; emission at 550 nm) using a FLUOstar Optima Fluorescence Plate Reader (BMG Labtech, UK). The plasmid pAJR70 containing the promoterless eGFP fusion was used as a negative control for background fluorescence. GraphPad Prism 5.0 (San Diego, CA, USA) was used to both plot and analyze the data. Experiments presented are the mean (±SEM) of three biological replicates.

### Transmission electron microscopy

Bacterial samples were fixed on ice with 1% paraformaldehyde/PBS for an hour and washed three times in PBS for 5 min each time. Sample suspension droplets (5 μl) were placed onto Formvar/Carbon nickel‐coated support grids, which had been pretreated with a 0.1% poly‐L‐lysine solution. Bacterial suspensions were left to settle for 20 min, the grids were floated sample on 0.05 M glycine/PBS droplets for 5 min and then rinsed three times with 3% BSA/PBS for 5 min. Bacterial samples were placed onto an α‐EspA (1:100 dilution) for an hour, washed six times on 3% BSA/PBS for 5 min each and then placed onto droplets of secondary GAR 10 nm gold for an hour. Samples were washed six times in 3% BSA/PBS for 5 min each, rinsed with PBS six times for 3 min, fixed on 1% glutaraldehyde/PBS for 5 min, washed six times in distilled water and then left to dry. Immunostained bacterial samples were viewed on a FEI Tecnai T20 TEM running at 200 kV and dm4/tiff images were captured using Gatan Digital Imaging software.

### Total RNA extraction and mRNA enrichment

Bacteria were cultured as described above in MEM‐HEPES before being mixed with two volumes of RNAprotect reagent (Qiagen, Valencia, CA, USA), incubated for 5 min at room temperature and harvested by centrifugation. Three biological replicates were used for each condition of the transcriptome analysis. Total RNA was extracted using an RNeasy kit (Qiagen) according to the manufacturers’ specifications. Genomic DNA was removed using TURBO DNase (Ambion, Carlsbad, CA, USA). Total RNA samples were enriched for mRNA using a MICROBexpress mRNA enrichment kit (Ambion). Integrity of enriched mRNA samples for sequencing were tested using an Agilent Bioanalyzer 2100 at the University of Glasgow Polyomics Facility.

### RNA‐sequencing and transcriptome analysis

cDNA synthesis, library generation and Illumina sequencing was performed at the Edinburgh Genomics facility on the Illumina HiSeq 2500 platform obtaining 100 bp single end reads. Samples of TUV 93–0 cultured in MEM‐HEPES with and without 50 μM RCZ20 were used to extract mRNA. Raw sequencing reads were assessed for quality using FastQC (Babraham Bioinformatics, Cambridge, UK) and trimmed where necessary using CLC Genomics Workbench (CLC Bio, Aarhus, Denmark). Sequence reads were then mapped to the EDL933 reference genome (NCBI NC_002655.2) allowing for 3 mismatches per read and at least 5 reads per open reading frame under all conditions tested. To determine differential expression between conditions, analysis was performed using the Empirical analysis of DGE tool in CLC Genomics Workbench, which implements the EdgeR Bioconductor tool (Robinson *et al*., 2010). Differentially expressed genes were identified by positive or negative absolute fold change and considered significant if the corrected *p*‐value was ≤ 0.05 (false‐discovery rate of 5%). Differentially expressed genes (DEGs) were grouped by biological function according to their designated gene ontology (GO) terms obtained from the EMBL‐EBI QuickGO database (https://www.ebi.ac.uk/QuickGO/). Bar charts summarizing the data were generated in GraphPad Prism version 5.0 (San Diego, CA, USA) and heatmaps of gene expression patterns were generated in Microsoft Excel. The raw sequence reads in this article have been deposited in the European Nucleotide Archive under the identifiers PRJEB17972 (treated with RCZ20) and the previous data set PRJEB12065 used as the untreated control for comparative purposes.

## Author Contributions

RM and AJR conceived, designed and coordinated the research. RZ, JPRC, AHU and KB performed the research and analyzed the data. RZ, JPRC, RM and AJR wrote the paper.

## Supporting information

Supporting Figures and Tables
