# Supplementary material for: Novel compounds targeting the enterohemorrhagic Escherichia coli type three secretion system reveal insights into mechanisms of secretion inhibition
Source: Mol Microbiol. 2017 Jun 14;105(4):606–19. doi: 10.1111/mmi.13719 (PMC5575525; doi:10.1111/mmi.13719)
Supplement: Supplementary file 1 — Supporting Figures and Tables [file MMI-105-606-s001.docx]

**Supporting information**

**Novel Compounds Targeting the Enterohaemorrhagic *Escherichia coli* Type Three Secretion System Reveal Insights into Mechanisms of Secretion Inhibition**

Riccardo Zambelloni^1^, James P.R. Connolly^1^, Alejandro Huerta Uribe^1^, Karl Burgess^1^, Rodolfo Marquez^2^ and Andrew J. Roe^1*^

Table of contents:

1. Supplementary materials and methods
2. Supplementary tables 1, 2, 3 and 4
3. Supplementary figures 1 and 2

**Supplementary Materials and Methods**

### **Chemical synthesis**

All chemicals used in this study were purchased from Sigma-Aldrich, Acros Organics, Fisher Scientific, Life Technologies, Agilent and NEB and used as sold. All reactions were performed under a dry Argon atmosphere and monitored using TLC plates on pre-coated silica Merck, visualised under UV light at 256 nm. Proton NMR spectra were recorded on Bruker AM-400 and carbon NMR spectra on a Bruker AM-500. All chemical shift (δ) were reported in parts per million (ppm) with the following symbols: s singlet, d doublet, t triplet, dd doublet of doublets, dt doublet of triplets, dq doublet of quartets, ddt doublet of doublet of triplets, dddd doublet of doublets of doublets of doublets, bs broad singlet, m multiplet. IR spectra were recorded on Shimadtzu FT-IR 8000S with neat samples. Uncorrected melting points were performed with a Stuart Scientific Melting Point SMP1. All flash chromatography columns were performed using Merck Silica.

### **General procedure for Friedel-Crafts acylation:**

The desired anhydride (47.77 mmol) was dissolved in dry DCE (100 mL) and anhydrous aluminium chloride (60.78 mmol) was added at 0 °C in one portion. After being stirred for 15 minutes, 1,3-dimethoxybenzene (43.42 mmol) was added dropwise at 0 °C through 30 minutes. The ice bath was removed and the reaction mixture was stirred at room temperature for 5 hours. Water (200 mL) was added carefully at 0 °C to quench the excess of aluminium chloride and the aqueous layer was extracted with DCM (3x100 mL), dried over Na_2_SO_4_, filtered and concentrated. The product was purified through flash column chromatography.



4-(2,4-Dimethoxy-phenyl)-4-oxo-butyric acid (2): The product was purified through flash column chromatography (2% methanol in DCM) to afford an off-white solid in 65% yield. m.p. 138 °C. ^1^H-NMR (CDCl_3_ 400 MHz) δ 7.81 (1H, d, J = 8.8 Hz), 6.45 (1H, dd, J = 8.8 Hz, 2.3 Hz), 6.38 (1H, d, J = 2.3 Hz), 3.83 (3H, s), 3.78 (3H, s), 3.22 (2H, t, J = 6.6 Hz), 2.66 (2H, t, J = 6.6 Hz). ^13^C-NMR (CDCl_3_ 126 MHz) δ 197.5, 178.7, 161.2, 155.6, 132.9, 108.3, 105.2, 98.3, 55.5, 55.4, 38.4, 28.7. IR (neat) ν_max_: 2878, 1606, 1498, 1100, 998 cm^-1^. HRMS (ESI) calcd for C_12_H_14_NaO_5_ [M+Na]^+^ 261.0733 m/z found 261.0728 m/z.



2-(2,4-Dimethoxy-benzoyl)-benzoic acid (3): The product was purified through flash column chromatography (5% methanol in DCM) to give an off-white solid in 54% yield. m.p. 134 °C. ^1^H-NMR (CDCl_3_ 400 MHz) δ 7.93 (1H, d, J = 6.7 Hz), 7.67 (1H, d, J = 8.1 Hz), 7.50 (1H, t, J = 6.7 Hz), 7.39 (1H, t, J = 7.5 Hz), 7.21 (1H, d, J = 7.4 Hz), 6.44 (1H, d, J = 8.0 Hz), 6.30 (1H, s), 3.77 (3H, s), 3.47 (3H, s). ^13^C-NMR (CDCl_3_ 126 MHz) δ 194.6, 170.9, 165.0, 161.3, 145.8, 133.9, 133.1, 130.2, 128.5, 127.4, 126.8, 120.0, 105.2, 98.6, 55.5, 55.4. IR (neat) ν_max_: 3001, 1721, 1595, 1261, 1122 cm^-1^. HRMS (ESI) calcd for C_16_H_13_O_5_ [M-H]^-^ 285.0768 m/z found 285.0755 m/z.

### **General procedure for hydrazine coupling:**

The desired carboxylic acid (**2** or **3**) (8.73 mmol) was suspended in absolute ethanol (25 mL) and hydrazine hydrate (17.50 mmol) was added via syringe. The reaction mixture was refluxed at 100 °C until total consumption of the starting material, monitored by TLC (normally 3-4 hours). The solvent was then removed and the product was dried under vacuum pump to give the desired product in excellent purity.



6-(2,4-Dimethoxy-phenyl)-4,5-dihydro-2H-pyridazin-3-one (4): The product was obtained as a yellowish solid in 96% yield in 2 hours. m.p. 121 °C. ^1^H-NMR (CDCl_3_ 400 MHz) δ 8.60 (1H, s), 7.33 (1H, d, J = 8.4 Hz), 6.44 (1H, dd, J = 8.4 Hz, 2.4 Hz), 6.40 (1H, d, J = 2.3 Hz), 3.76 (6H, s), 2.86 (2H, t, J = 8.2 Hz), 2.44 (2H, t, J = 8.4 Hz). ^13^C-NMR (CDCl_3_ 126 MHz) δ 168.2, 162.2, 158.6, 153.5, 130.0, 118.9, 104.8, 98.7, 55.5, 55.4, 26.6, 25.7. IR (neat) ν_max_: 3274, 2935, 1672, 1334, 1207 cm^-1^. HRMS (ESI) calcd for C_12_H_17_N_2_O_2_ [M+H]^+^ 221.1285 m/z found 221.1282 m/z.



4-(2,4-Dimethoxy-phenyl)-2H-phthalazin-1-one (5): The product was obtained by suction filtration as an off-white solid in 85% yield in 4 hours. m.p. 256 °C. ^1^H-NMR (CDCl_3_ 400 MHz) δ 10.43 (1H, s), 8.40 (1H, dd, J = 7.5 Hz, 1.8 Hz), 7.70 - 7.65 (2H, m), 7.32 (1H, dd, J = 7.6 Hz, 1.8 Hz), 7.21 (1H, d, J = 8.2 Hz), 6.55 (1H, dd, J = 8.2 Hz, 2.3 Hz), 6.52 (1H, d, J = 2.3 Hz), 3.81 (3H, s), 3.63 (3H, s). ^13^C-NMR (CDCl_3_ 126 MHz) δ 162.0, 160.4, 158.6, 146.6, 133.0, 131.8, 131.2, 130.8, 127.9, 127.4, 126.3, 116.7, 104.8, 98.8, 55.5, 55.4. IR (neat) ν_max_: 3142, 2922, 1676, 1208, 1157 cm^-1^. HRMS (ESI) calcd for C_16_H_14_N_2_NaO_3_ [M+Na]^+^ 305.0897 m/z found 305.0895 m/z.

### **General procedure for LiAlH_4_ reduction** (Combs, Reese, & Phillips, 1995)**:**

LiAlH_4_ (6.40 mmol) was suspended in dry THF (10 mL) and a solution of the desired amide (**4** or **5**) (2.13 mmol) in dry THF (10 mL) was added dropwise at room temperature. The reaction mixture was refluxed at 80 °C for 5 hours. Water (20 mL) was added very carefully at 0 °C to quench the excess of LiAlH_4_ and the aqueous layer was extracted with ethyl acetate (3x30 mL), dried over Na_2_SO_4_, filtered and concentrated. The desired product was pure enough and it was used in the next step without any further purification.



3-(2,4-Dimethoxy-phenyl)-1,4,5,6-tetrahydro-pyridazine (6): The product was obtained as a yellow thick oil in 81% yield and it was used without any further purification. ^1^H-NMR (CDCl_3_ 400 MHz) δ 7.30 (1H, d, J = 8.4 Hz), 6.50 (1H, dd, J = 8.3 Hz, 2.4 Hz), 6.47 (1H, d, J = 2.4 Hz), 3.84 (3H, s), 3.83 (3H, s), 3.18 (2H, t, J = 5.5 Hz), 2.60 (2H, t, J = 6.8 Hz), 2.03 (2H, dddd, J = 6.7 Hz, 6.7 Hz, 5.5 Hz, 5.5 Hz). ^13^C-NMR (CDCl_3_ 126 MHz) δ 162.0, 160.4, 158.6, 146.6, 116.7, 104.8, 98.8, 55.5, 55.4, 45.6, 25.5, 19.1. IR (neat) ν_max_: 3366, 2935, 1609, 1206, 919 cm^-1^. HRMS (ESI) calcd for C_12_H_14_N_2_NaO_3_ [M+Na]^+^ 257.0897 m/z found 257.0897 m/z.



4-(2,4-Dimethoxy-phenyl)-1,2-dihydro-phthalazine (7): The product was obtained as a yellowish solid in 94% yield and with excellent purity. m.p. 124 °C. ^1^H-NMR (CDCl_3_ 400 MHz) δ 7.37 (1H, d, J = 8.3 Hz), 7.34 (1H, t, J = 7.5 Hz), 7.21 (1H, t, J = 7.7 Hz), 7.15 (1H, d, J = 7.5 Hz), 6.89 (1H, d, J = 7.7 Hz), 6.60 (1H, dd, J = 8.3 Hz, 2.3 Hz), 6.56 (1H, d, J = 2.3 Hz), 6.06 (1H, s), 4.31 (2H, s), 3.88 (3H, s), 3,72 (3H, s). ^13^C-NMR (CDCl_3_ 126 MHz) δ 161.2, 158.8, 147.8, 131.5, 131.4, 129.8, 127.5, 127.4, 124.8, 118.6, 104.6, 98.8, 55.5, 55.4, 45.6. IR (neat) ν_max_: 3364, 2936, 1608, 1207, 826 cm^-1^. HRMS (ESI) calcd for C_16_H_17_N_2_O_2_ [M+H]^+^ 269.1285 m/z found 269.1279 m/z.

### **General procedure for acylation/sulphonylation:**

The desired amine derivatives (**6** or **7**) (1.63 mmol) was dissolved in dry DCM (10 mL) and TEA (3.42 mmol) and DMAP (0.16 mmol) were added subsequently. After 5 minutes, the desired acid/sulfonyl chloride (1.79 mmol) was added and the reaction mixture was stirred at room temperature until the starting material was no longer detectable by TLC (2-16 hours). The solvent was then removed and the crude material was purified through direct flash column chromatography.



[3-(2,4-Dimethoxy-phenyl)-5,6-dihydro-4H-pyridazin-1-yl]-(4-nitro-phenyl)-methanone (RCZ09): The product was purified with direct flash column chromatography (from 10% to 20% ethyl acetate in petroleum ether) to afford a yellow solid in 60% yield. m.p. 152 °C. ^1^H-NMR (CDCl_3_ 400 MHz) δ 8.13 (2H, d, J = 8.8 Hz), 7.78 (2H, d, J = 8.9 Hz), 7.06 (1H, d, J = 8.2 Hz), 6.35 (1H, d, J = 2.4 Hz), 6.34 (1H, dd, J = 8.3 Hz, 2.4 Hz), 3.93 (2H, t, J = 5.9 Hz), 3.72 (3H, s), 3.71 (3H, s), 2.65 (2H, t, J = 6.3 Hz), 1.95 (2H, dddd, J = 6.3 Hz, 6.3 Hz, 5.7 Hz, 5.7 Hz). ^13^C-NMR (CDCl_3_ 126 MHz) δ 168.2, 161.7, 158.8, 151.2, 148.2, 141.9, 130.5, 129.7, 122.5, 120.3, 104.6, 98.8, 55.4, 55.3, 40.1, 26.8, 17.6. IR (neat) ν_max_: 2838, 1647, 1520, 1344, 1160 cm^-1^. HRMS (ESI) calcd for C_19_H_19_N_3_NaO_5_ [M+Na]^+^ 392.1217 m/z found 392.1200 m/z.



3-(2,4-Dimethoxy-phenyl)-1-(4-nitro-benzenesulfonyl)-1,4,5,6-tetrahydro-pyridazine (RCZ11): The product was purified with direct flash column chromatography (20% ethyl acetate in petroleum ether) to afford a brownish solid in 67% yield. m.p. 168 °C. ^1^H-NMR (CDCl_3_ 400 MHz) δ 8.27 (2H, d, J = 9.0 Hz), 8.04 (2H, d, J = 8.9 Hz), 7.15 (1H, d, J = 8.5 Hz), 6.40 (1H, dd, J = 8.4 Hz, 2.3 Hz), 6.34 (1H, d, J = 2.3 Hz), 3.75 (3H, s), 3.66 (3H, s), 3.39 (2H, t, J = 5.6 Hz), 2.49 (2H, t, J = 6.6 Hz), 1.97 (2H, dddd, J = 6.6 Hz, 6.6 Hz, 5.6 Hz, 5.6 Hz). ^13^C-NMR (CDCl_3_ 126 MHz) δ 161.8, 158.6, 154.7, 150.2, 142.0, 130.4, 129.7, 123.8, 120.1, 104.6, 98.7, 55.4, 55.3, 43.1, 25.1, 19.0. IR (neat) ν_max_: 2925, 1606, 1527, 1308, 1172 cm^-1^. HRMS (ESI) calcd for C_18_H_19_N_3_NaO_6_S [M+Na]^+^ 428.0887 m/z found 428.0892 m/z.



[4-(2,4-Dimethoxy-phenyl)-1H-phthalazin-2-yl]-(4-nitro-phenyl)-methanone (RCZ17): The product was purified with direct flash column chromatography (20% ethyl acetate in petroleum ether) to give a bright yellow solid in 54% yield. m.p. 183 °C ^1^H-NMR (CDCl_3_ 400 MHz) δ 8.26 (2H, d, J = 9.1 Hz), 7.96 (2H, d, J = 9.1 Hz), 7.50 (1H, t, J = 7.5 Hz), 7.33 (1H, t, J = 7.5 Hz), 7.32 (1H, d, J = 7.8 Hz), 7.20 (1H, d, J = 8.2 Hz), 7.06 (1H, d, J = 7.8 Hz), 6.58 (1H, dd, J = 8.1 Hz, 2.3 Hz), 6.55 (1H, d, J = 2.3 Hz), 5.20 (2H, s), 3.87 (3H, s), 3.75 (3H, s). ^13^C-NMR (CDCl_3_ 126 MHz) δ 168.5, 162.1, 158.9, 151.8, 148.7, 140.6, 131.6, 131.2, 131.0, 130.3, 128.0, 127.3, 126.8, 125.9, 122.6, 116.6, 104.8, 98.9, 55.5, 55.4, 42.7. IR (neat) ν_max_: 2926, 1651, 1520, 1344, 1209 cm^-1^. HRMS (ESI) calcd for C_23_H_19_N_3_NaO_5_ [M+Na]^+^ 440.1217 m/z found 440.1202 m/z.



4-(2,4-Dimethoxy-phenyl)-2-(4-nitro-benzenesulfonyl)-1,2-dihydro-phthalazine (RCZ19): The product was purified by direct flash column chromatography (20% ethyl acetate in petroleum ether) to afford a bright yellow solid in 75% yield. m.p. 186 °C. ^1^H-NMR (CDCl_3_ 400 MHz) δ 8.38 (2H, d, J = 8.9 Hz), 8.21 (2H, d, J = 8.9 Hz), 7.44 (1H, t, J = 7.4 Hz), 7.42 (1H, d, J = 8.4 Hz), 7.27 (1H, t, J = 7.4 Hz), 7.25 (1H, d, J = 7.4 Hz), 6.98 (1H, d, J = 7.4 Hz), 6.62 (1H, dd, J = 8.4 Hz, 2.4 Hz), 6.51 (1H, d, J = 2.4 Hz), 4.53 (2H, s), 3.90 (3H, s), 3.60 (3H, s). ^13^C-NMR (CDCl_3_ 126 MHz) δ 162.3, 158.8, 154.3, 150.4, 141.3, 132.0, 131.4, 130.3, 130.0, 128.2, 126.6, 126.1, 125.5, 123.9, 116.6, 104.9, 98.8, 55.5, 55.3, 45.8. IR (neat) ν_max_: 2936, 1608, 1348, 1177, 738 cm^-1^. HRMS (ESI) calcd for C_22_H_20_N_3_O_6_S [M+H]^+^ 454.0995 m/z found 454.0999 m/z.

### **General procedure for demethylation:**

The corresponding dimethoxy analogue (RCZ09, RCZ11, RCZ17 or RCZ19) (0.24 mmol) was dissolved in dry DCM (6 mL) and a 1 M solution of BBr_3_ in DCM (2.4 mmol) was added dropwise at 0 °C. The reaction mixture was then heated up to 40 °C until starting material consumption (3-36 hours), monitored by TLC. After cooling to room temperature, the excess of BBr_3_ was quenched with a careful addition of water (20 mL) and the layers were separated. The aqueous layer was then washed with DCM (2x20 mL) and the combined organic layers were dried over Na_2_SO_4_, filtered and concentrated. The crude was purified through flash column chromatography to give the desired product.



[3-(2,4-Dihydroxy-phenyl)-5,6-dihydro-4H-pyridazin-1-yl]-(4-nitro-phenyl)-methanone (RCZ10): The product was purified through flash column chromatography (1% methanol in DCM) to give a yellow solid in 55% yield. m.p. 231 °C. ^1^H-NMR (DMSO 400 MHz) δ 10.60 (1H, s), 9.83 (1H, s), 8.33 (2H, d, J = 8.6 Hz), 7.79 (2H, d, J = 8.6 Hz), 7.33 (1H, d, J = 8.8 Hz), 6.27 (1H, dd, J = 8.8 Hz, 2.2 Hz), 6.03 (1H, d, J = 2.2 Hz), 3.91 (2H, t, J = 5.7 Hz), 2.76 (2H, t, J = 6.9 Hz), 2.04 (2H, dddd, J = 6.8 Hz, 6.8 Hz, 5.7 Hz, 5.7 Hz). ^13^C-NMR (DMSO 126 MHz) δ 167.1, 160.1, 158.7, 153.8, 147.8, 142.2, 128.9, 128.8, 123.6, 110.9, 107.1, 102.7, 44.1, 22.7, 16.8. IR (neat) ν_max_: 3385, 2359, 1627, 1518, 1349 cm^-1^. HRMS (ESI) calcd for C_17_H_15_N_3_NaO_5_ [M+Na]^+^ 364.0904 m/z found 364.0890 m/z.





4-[1-(4-Nitro-benzenesulfonyl)-1,4,5,6-tetrahydro-pyridazin-3-yl]-benzene-1,3-diol (RCZ12): The product was run through flash column chromatography (50% ethyl acetate in petroleum ether) to afford a bright yellow oil/yellow foam in 15%. ^1^H-NMR (CDCl_3_ 400 MHz) δ 11.79 (1H, s), 8.30 (2H, d, J = 9.0 Hz), 8.01 (2H, d, J = 9.0 Hz), 7.08 (1H, d, J = 8.7 Hz), 6.36 (1H, d, J = 2.5 Hz), 6.27 (1H, dd, J = 8.5 Hz, 2.5 Hz), 4.98 (1H, s), 3.40 (2H, t, J = 6.9 Hz), 2.51 (2H, t, J = 6.7 Hz), 2.11 (2H, dddd, J = 6.9 Hz, 6.9 Hz, 6.7 Hz, 6.7 Hz). ^13^C-NMR (CDCl_3_ 126 MHz) δ 161.8, 158.6, 154.7, 150.2, 142.0, 130.4, 129.7, 123.8, 120.1, 104.6, 98.7, 43.1, 25.1, 19.0. IR (neat) ν_max_: 3431, 2924, 1627, 1530, 1349 cm^-1^. HRMS (ESI) calcd for C_16_H_15_N_3_O_6_S [M+H]^+^ 378.0743 m/z found 378.0723 m/z.



[4-(2,4-Dihydroxy-phenyl)-1H-phthalazin-2-yl]-(4-nitro-phenyl)-methanone (RCZ18): The product was purified through flash column chromatography (1% methanol in DCM) to give an intense yellow solid in 74% yield. m.p. 253 °C. ^1^H-NMR (DMSO 400 MHz) δ 9.60 (1H, s), 9.51 (1H, s), 8.28 (2H, d, J = 8.7 Hz), 7.93 (2H, d, J = 8.9 Hz), 7.53 (1H, t, J = 7.6 Hz), 7.49 (1H, d, J = 7.2 Hz), 7.36 (1H, t, J = 7.7 Hz), 7.07 (1H, d, J = 7.2 Hz), 7.05 (1H, d, J = 8.3 Hz), 6.35 (1H, d, J = 2.3 Hz), 6.29 (1H, dd, J = 8.3 Hz, 2.3 Hz), 5.09 (2H, s). ^13^C-NMR (CDCl_3_ 126 MHz) δ 167.8, 159.5, 156.8, 151.8, 148.0, 140.8, 131.4, 131.2, 130.7, 130.4, 127.9, 126.7, 126.1, 125.3, 122.7, 112.7, 106.8, 102.5, 42.1. IR (neat) ν_max_: 2931, 2355, 1622, 1348, 1103 cm^-1^. HRMS (ESI) calcd for C_21_H_14_N_3_O_5_ [M-H]^-^ 388.0939 m/z found 388.0931 m/z.



4-[3-(4-Nitro-benzenesulfonyl)-3,4-dihydro-phthalazin-1-yl]-benzene-1,3-diol (RCZ20): The product was purified through flash column chromatography (1% methanol in DCM) to afford a yellow foam in 25% yield. ^1^H-NMR (CDCl_3_ 400 MHz) δ 10.34 (1H, s), 8.33 (2H, d, J = 8.7 Hz), 8.09 (2H, d, J = 8.7 Hz), 7.48 (1H, t, J = 7.5 Hz), 7.43 (1H, d, J = 7.5 Hz), 7.35 (1H, t, J = 7.7 Hz), 7.26 (1H, d, J = 7.7 Hz), 7.18 (1H, d, 8.5 Hz), 6.50 (1H, d, 2.3 Hz), 6.33 (1H, dd, J = 8.5 Hz, 2.3 Hz), 5.10 (1H, s), 4.29 (2H, s). ^13^C-NMR (CDCl_3_ 126 MHz) δ 161.5, 160.3, 157.7, 152.0, 140.9, 134.0, 133.3, 133.0, 131.9, 129.3, 128.6, 127.4, 125.5, 125.4, 110.5, 108.0, 104.6, 47.3. IR (neat) ν_max_: 3122, 2926, 2363, 1356, 652 cm^-1^. HRMS (ESI) calcd for C_20_H_15_N_3_NaO_6_S [M+Na]^+^ 448.0574 m/z found 448.0563 m/z.

### **General procedure for allylation:**

**RCZ12** or **RCZ20** (0.26 mmol) were dissolved in dry DMF (12 mL) and anhydrous potassium carbonate (0.78 mmol) was added. After 15 minutes, allyl bromide (1.06 mmol) was added slowly to the reaction, which was stirred at room temperature overnight. Water (10 mL) was added and extracted with ether (3x15 mL). The organic phase was then washed with brine (5x30 mL), dried over Na_2_SO_4_, filtered and concentrated. The product thus obtained was used in the next step without further purification.



3-(2,4-Bis-allyloxy-phenyl)-1-(4-nitro-benzenesulfonyl)-1,4,5,6-tetrahydro-pyridazine (8): The product was obtained as a yellow oil (106 mg, 0.231 mmol, 89%). ^1^H-NMR (CDCl_3_ 400 MHz) δ 8.27 (2H, d, J = 8.9 Hz), 8.03 (2H, d, J = 8.9 Hz), 7.14 (1H, d, J = 8.4 Hz), 6.41 (1H, dd, J = 8.4 Hz, 2.3 Hz), 6.36 (1H, d, J = 2.2 Hz), 5.97 (1H, ddt, J = 17.2 Hz, 10.6 Hz, 5.2 Hz), 5.87 (1H, ddt, J = 17.2 Hz, 10.1 Hz, 5.3 Hz), 5.35 (1H, dd, J = 17.2 Hz, 1.5 Hz), 5.23 (1H, dd, J = 9.8 Hz, 1.2 Hz), 5.22 (1H, dd, J = 15.6 Hz, 1.3 Hz), 5.15 (1H, dd, J = 10.4 Hz, 1.2 Hz), 4.46 (2H, dt, J = 5.4 Hz, 1.4 Hz), 4.39 (2H, dt, J = 5.2 Hz, 1.5 Hz), 3.40 (2H, t, J = 5.6 Hz), 2.51 (2H, t, J = 6.6 Hz), 1.97 (2H, dddd, J = 6.6 Hz, 6.6 Hz, 5.7 Hz, 5.7 Hz). ^13^C-NMR (CDCl_3_ 126 MHz) δ 160.7, 157.4, 154.7, 150.3, 142.1, 132.8, 132.7, 130.3, 129.7, 123.8, 118.2, 117.7, 105.8, 100.6, 69.1, 68.9, 43.1, 26.0, 18.9. IR (neat) ν_max_: 2359, 1607, 1529, 1349, 930 cm^-1^. HRMS (ESI) calcd for C_22_H_23_N_3_NaO_6_S [M+Na]^+^ 480.1216 m/z found 480.1231 m/z.



4-(2,4-Bis-allyloxy-phenyl)-2-(4-nitro-benzenesulfonyl)-1,2-dihydro-phthalazine (9): The product was obtained as a yellow oil (89 mg, 0.176 mmol, 99%). ^1^H-NMR (CDCl_3_ 400 MHz) δ 8.27 (2H, d, J = 9.0 Hz), 8.10 (2H, d, J = 9.0 Hz), 7.35 (1H, d, J = 8.5 Hz), 7.33 (1H, t, J = 7.7 Hz), 7.15 (2H, m), 6.92 (1H, d, J = 7.6 Hz), 6.54 (1H, dd, J = 8.5 Hz, 2.3 Hz), 6.42 (1H, d, J = 2.3 Hz), 6.01 (1H, ddt, J = 17.2 Hz, 10.6 Hz, 5.4 Hz), 5.51 (1H, ddt, J = 17.1 Hz, 10.6 Hz, 4.4 Hz), 5.38 (1H, dq, J = 17.1 Hz, 1.5 Hz), 5.27 (1H, dq, J = 10.5 Hz, 1.5 Hz), 4.83 (1H, dq, J = 10.8 Hz, 1.6 Hz), 4.69 (1H, dq, J = 17.2 Hz, 1.6 Hz), 4.51 (2H, dt, J = 5.2 Hz, 1.5 Hz), 4.41 (2H, s), 4.24 (2H, dt, J = 4.4 Hz, 1.5 Hz). ^13^C-NMR (CDCl_3_ 126 MHz) δ 161.2, 157.7, 157.6, 148.4, 141.3, 132.9, 132.3, 131.9, 131.8, 131.4, 130.3, 130.1, 128.2, 127.5, 126.7, 125.4, 123.9, 118.0, 116.3, 106.1, 100.6, 69.0, 68.6, 45.8. IR (neat) ν_max_: 2359, 1608, 1356, 1171, 1029 cm^-1^. HRMS (ESI) calcd for C_26_H_23_N_3_NaO_6_S [M+Na]^+^ 528.1200 m/z found 528.1186 m/z.

### **General procedure for nitro reduction:**

The desired bis-allyloxy derivative (**8** or **9**) (0.23 mmol) was dissolved in absolute ethanol (12 mL) and iron powder (0.92 mmol) was added. A saturated solution of ammonium chloride (4 mL) was subsequently added until a white precipitate formed. The reaction mixture was then refluxed at 80 °C for 16 hours. After cooling to room temperature, the red/brown solution was filtered through a short pad of Celite and the residue washed with ethanol (25 mL) and ethyl acetate (25 mL). The solution was concentrated to dryness under reduced pressure, a mixture of DCM/water (30 mL) was added to the solid and the phases were separated. The aqueous phase was back-washed with DCM (2x15 mL) and the combined organic phases were dried over Na_2_SO_4_, filtered and concentrated. The product thus obtained was used in the next step without further purification.



4-[3-(2,4-Bis-allyloxy-phenyl)-5,6-dihydro-4H-pyridazine-1-sulfonyl]-phenylamine (10): The product was afforded as a yellow/brown oil (75 mg, 0.175 mmol, 85%).^1^H-NMR (CDCl_3_ 400 MHz) δ 7.63 (2H, d, J = 8.7 Hz), 7.16 (1H, d, J = 8.5 Hz), 6.61 (2H, d, J = 8.7 Hz), 6.38 (1H, dd, J = 8.5 Hz, 2.3 Hz), 6.35 (1H, d, J = 2.3 Hz), 6.01 - 5,86 (2H, m), 5.33 (1H, dd, J = 17.2 Hz, 1.6 Hz), 5.25 (1H, dd, J = 16.4 Hz, 1.4 Hz), 5.22 (1H, dd, J = 10.4 Hz, 1.4 Hz), 5.17 (1H, dd, J = 10.5 Hz, 1.5 Hz), 4.45 (2H, dt, J = 6.5 Hz, 1.4 Hz), 4.39 (2H, dt, J = 5.0 Hz, 1.4 Hz), 4.03 (2H, s), 3.29 (2H, t, J = 5.6 Hz), 2.48 (2H, t, J = 6.7 Hz), 1.94 (2H, dddd, J = 6.7 Hz, 6.7 Hz, 5.6 Hz, 5.6 Hz). ^13^C-NMR (CDCl_3_ 126 MHz) δ 160.3, 157.6, 153.3, 150.6, 133.0, 132.9, 130.7, 130.6, 121.4, 117.8, 117.6, 113.9, 113.7, 105.9, 100.6, 69.3, 68.9, 43.1, 25.8, 19.2. IR (neat) ν_max_: 3451, 2390, 1616, 1189, 1054 cm^-1^. HRMS (ESI) calcd for C_22_H_25_N_3_NaO_4_S [M+Na]^+^ 450.1554 m/z found 450.1576 m/z.



4-[4-(2,4-Bis-allyloxy-phenyl)-1H-phthalazine-2-sulfonyl]-phenylamine (11): The product was afforded as a yellow/brown oil (81 mg, 0.170 mmol, 99%). ^1^H-NMR (CDCl_3_ 400 MHz) δ 7.67 (2H, d, J = 8.6 Hz), 7.37 (1H, d, J = 8.3 Hz), 7.28 (1H, t, J = 7.4 Hz), 7.17 - 7.11 (2H, m), 6.89 (1H, d, J = 7.6 Hz), 6.58 (2H, d, J = 8.7 Hz), 6.52 (1H, dd, J = 8.3 Hz, 2.3 Hz), 6.42 (1H, d, J = 2.3 Hz), 6.01 (1H, ddt, J = 17.1 Hz, 10.5 Hz, 5.6 Hz), 5.54 (1H, ddt, J = 17.2 Hz, 10.6 Hz, 4.8 Hz), 5.37 (1H, dd, J = 17.2 Hz, 1.4 Hz), 5.25 (1H, dd, J = 10.5 Hz, 1.4 Hz), 4.85 (1H, dd, J = 10.6 Hz, 1.6 Hz), 4.75 (1H, dd, J = 17.2 Hz, 1.5 Hz), 4.50 (2H, dt, J = 5.4 Hz, 1.4 Hz), 4.30 (2H, s), 4.25 (2H, dt, J = 4.5 Hz, 1.4 Hz), 4.03 (2H, s). ^13^C-NMR (CDCl_3_ 126 MHz) δ 160.8, 157.7, 152.7, 150.9, 133.0, 132.5, 132.1, 131.2, 130.9, 130.8, 127.7, 126.6, 126.3, 125.3, 118.0, 117.9, 116.4, 113.8, 106.1, 100.8, 69.0, 68.8, 45.7. IR (neat) ν_max_: 3374, 2359, 1623, 1594, 1163 cm^-1^. HRMS (ESI) calcd for C_26_H_25_N_3_NaO_4_S [M+Na]^+^ 498.1458 m/z found 498.1444 m/z.

### **General procedure for hex-5-ynoyl chloride coupling:**



Hex-5-ynoyl chloride: Hex-5-ynoic acid (0.495 mL, 4.45 mmol) was dissolved in dry DCM (20 mL) and two drops of dry DMF were added. Oxalyl chloride (0.421 mL, 4.90 mmol) was added dropwise at 0 °C through 10 minutes. The reaction mixture was then allowed to stir at room temperature until the carbon dioxide bubbling ceased (2 hours). The solution was then concentrated under reduced pressure to afford a red oil which was used immediately for the next step.

The desired aniline derivative (**10** or **11**) (0.60 mmol) was dissolved in dry DCM (10 mL) and anhydrous pyridine (0.64 mmol) was added. After 10 minutes, a freshly prepared solution of hex-5-ynoyl chloride (0.32 mmol) in dry DCM (3 mL) was added slowly. The reaction mixture was allowed to stir at room temperature for 3 hours. The solvent was evaporated off under reduced pressure and the product was purified through direct flash column chromatography.



Hex-5-ynoic acid {4-[3-(2,4-bis-allyloxy-phenyl)-5,6-dihydro-4H-pyridazine-1-sulfonyl]-phenyl}-amide (12): The product was purified through direct flash column chromatography (30% ethyl acetate in petroleum ether) to afford a colourless oil (75 mg, 0.143 mmol, 89%).^1^H-NMR (CDCl_3_ 400 MHz) δ 7.90 (2H, d, J = 8.6 Hz), 7.69 (2H, d, J = 8.6 Hz), 7.39 (1H, s), 7.23 (1H, d, J = 8.5 Hz), 6.48 (1H, dd, J = 8.5 Hz, 2.3 Hz), 6.44 (1H, d, J = 2.3 Hz), 6.06 (1H, ddt, J = 15.8 Hz, 10.6 Hz, 5.6 Hz), 5.98 (1H, ddt, J = 15.9 Hz, 10.8 Hz, 5.6 Hz), 5.42 (1H, dd, J = 17.2 Hz, 1.5 Hz), 5.33 (1H, dd, J = 17.2 Hz, 1.5 Hz), 5.30 (1H, dd, J = 10.6 Hz, 1.5 Hz), 5.25 (1H, dd, J = 10.5 Hz, 1.3 Hz), 4.55 (2H, dt, J = 5.5 Hz, 1.3 Hz), 4.48 (2H, dt, J = 5.2 Hz, 1.3 Hz), 3.42 (2H, t, J = 5.6 Hz), 2.67 (2H, t, J = 7.4 Hz), 2.54 (2H, t, J = 7.3 Hz), 2.05 - 1.95 (5H, m), 1.89 (2H, dddd, J = 7.3 Hz, 7.3 Hz, 5.9 Hz, 5.9 Hz). ^13^C-NMR (CDCl_3_ 126 MHz) δ 166.8, 160.7, 160.4, 157.8, 153.8, 149.4, 133.0, 132.8, 130.6, 129.8, 118.9, 117.9, 117.6, 105.9, 100.6, 91.1, 69.2, 68.9, 60.5, 43.1, 31.8, 25.9, 23.6, 23.3, 17.7. IR (neat) ν_max_: 3209, 2357, 1701, 1590, 1166 cm^-1^. HRMS (ESI) calcd for C_28_H_31_N_3_NaO_5_S [M+Na]^+^ 544.1919 m/z found 544.1922 m/z.



Hex-5-ynoic acid {4-[4-(2,4-bis-allyloxy-phenyl)-1H-phthalazine-2-sulfonyl]-phenyl}-amide (13): The product was purified through direct flash column chromatography (30% ethyl acetate in petroleum ether) to afford a colourless oil (61 mg, 0.107 mmol, 73%). ^1^H-NMR (CDCl_3_ 400 MHz) δ 7.94 (2H, d, J = 8.8 Hz), 7.68 (2H, d, J = 8.8 Hz), 7.51 (1H, s), 7.45 (1H, d, J = 8.1 Hz), 7.40 (1H, t, J = 7.2 Hz), 7.23 (2H, m), 7.00 (1H, d, J = 7.2 Hz), 6.62 (1H, dd, J = 8.3 Hz, 2.1 Hz), 6.51 (1H, d, J = 2.1 Hz), 6.11 (1H, ddt, J = 17.2 Hz, 10.6 Hz, 5.4 Hz), 5.62 (1H, ddt, J = 17.2 Hz, 10.5 Hz, 4.7 Hz), 5.48 (1H, dq, J = 17.3 Hz, 1.6 Hz), 5.36 (1H, dq, J = 10.5 Hz, 1.4 Hz), 4.94 (1H, dq, J = 10.6 Hz, 1.6 Hz), 4.82 (1H, dq, J = 17.2 Hz, 1.6 Hz), 4.60 (2H, dt, J = 5.6 Hz, 1.4 Hz), 4.45 (2H, s), 4.34 (2H, dt, J = 4.7 Hz, 1.6 Hz), 2.57 (2H, t, J = 7.2 Hz), 2.34 (2H, dt, J = 6.7 Hz, 2.7 Hz), 2.03 (1H, t, J = 2.6 Hz), 1.96 (2H, quint., J = 7.0 Hz). ^13^C-NMR (CDCl_3_ 126 MHz) δ 170.9, 160.9, 157.7, 153.5, 142.4, 133.0, 132.4, 132.0, 131.1, 130.6, 130.3, 130.0, 127.9, 126.5, 126.4, 125.3, 119.0, 118.0, 117.6, 116.4, 106.0, 100.7, 83.2, 69.5, 69.0, 68.7, 45.8, 35.9, 23.6, 17.7. IR (neat) ν_max_: 3298, 2924, 2360, 1606, 1166 cm^-1^. HRMS (ESI) calcd for C_32_H_31_N_3_NaO_5_S [M+Na]^+^ 592.1877 m/z found 592.1854 m/z.



(+)-Biotin N-succinimidyl ester (Biotin-NHS) (W. L. Yu, Guizzunti, Foley, Burkart, & La Clair, 2010): (+)-Biotin (600 mg, 2.45 mmol) was dissolved in dry DMF (10 mL) and EDC (370 mg, 3.68 mmol) and NHS (424 mg, 3.68 mmol) were added subsequently. The reaction mixture was allowed to stir at room temperature overnight. The solution was then poured into ice and the white precipitate formed was filtered by suction filtration. The solid thus obtained was dried under high vacuum to afford an off-white powder (520 mg, 1.524 mmol, 62%), which matched all the analytical data of previously reported characterisation (W. L. Yu et al., 2010) ^1^H-NMR (MeOD 400 MHz) δ 4.52 (1H, dd, J = 8.0 Hz, 5.1 Hz), 4.35 (1H, dd, J = 7.8 Hz, 4.9 Hz), 3.27 - 3.22 (1H, m), 2.96 (1H, dd, J = 12.5 Hz, 4.7 Hz), 2.86 (4H, s), 2.73 (1H, d, J = 12.5 Hz), 2.69 (2H, t, J = 6.8 Hz), 1.86 - 1.78 (3H, m), 1.68 - 1.56 (3H, m). ^13^C-NMR (MeOD 500 MHz) δ, 170.6, 169.1, 163.1, 61.8, 59.7, 55.6, 31.4, 28.9, 28.1, 25.0, 24.1.



3-Azidopropylamine (Zabrodski, Baskin, Kaniraj, & Maayan, 2014): 3-Chloropropylamine hydrochloride (1.12 g, 8.62 mmol) was dissolved in water (25 mL) and sodium azide (1.68 g, 25.8 mmol) was added at room temperature. The reaction mixture was stirred at 80 °C overnight and then sodium hydroxide (pellets) (5 g) was added to basify the solution. The basic aqueous layer was extracted with diethyl ether (3x30 mL) and the organic phase was dried over Na_2_SO_4_, filtered and concentrated, to afford 3-azidopropylamine as a yellowish liquid (450 mg, 4.510 mmol, 52%). ^1^H-NMR (CDCl_3_ 400 MHz) δ 3.30 (2H, t, J = 6.4 Hz), 2.73 (2H, t, J = 6.4 Hz), 1.66 (2H, quint., J = 6.4 Hz). ^13^C-NMR (CDCl_3_ 500 MHz) δ 49.1, 39.3, 32.4.



5-(2-Oxo-hexahydro-thieno[3,4-d]imidazol-4-yl)-pentanoic acid (3-azido-propyl)-amide (Biotin-N_3_): 3-Azidopropylamine (112 mg, 1.12 mmol) was dissolved in dry methanol (8 mL). TEA (0.234 mL, 1.68 mmol) was added at room temperature and then (+)-biotin N-succinimidyl ester (**Biotin-NHS**) (200 mg, 0.56 mmol) was added one pot. The reaction mixture was stirred at room temperature for 24 hours. The solvent was removed under reduced pressure and the residue was directly chromatographed (from 0% to 10% methanol in ethyl acetate) to afford a white solid (185 mg, 0.567 mmol, 91%). ^1^H-NMR (MeOD 400 MHz) δ 4.50 (1H, dd, J = 7.8 Hz, 4.9 Hz), 4.32 (1H, dd, J = 8.0 Hz, 4.5 Hz), 3.37 (2H, t, J = 6.7 Hz), 3.26 (2H, t, J = 6.7 Hz), 3.22 - 3.19 (1H, m), 2.94 (1H, dd, J = 12.6 Hz, 4.9 Hz), 2.72 (1H, d, J = 12.6 Hz), 2.22 (2H, t, J = 7.5 Hz), 1.80 - 1.58 (6H, m), 1.45 (2H, quint., J = 7.5 Hz). ^13^C-NMR (MeOD 500 MHz) δ 175.2, 172.6, 61.8, 59.9, 55.3, 51.3, 39.9, 38.2, 35.6, 28.7, 28.0, 25.4, 24.7. IR (neat) ν_max_: 3025, 2359, 2100, 1678, 1465 cm^-1^. HRMS (ESI) calcd for C_13_H_22_N_6_NaO_2_S [M+Na]^+^ 349.1417 m/z found 349.1415 m/z.

### **General procedure for click reaction** (F. Yu et al., 2013)**:**

Oxo-hexahydro-thieno[3,4-d]imidazol-4-yl)-pentanoic acid (3-azido-propyl)-amide (Biotin-N_3_) (0.09 mmol) and the desired alkyne derivative (**12** or **13**) (0.11 mmol) were dissolved in a 3/1 mixture of THF/H_2_O (4 mL) and copper(II)sulfate (0.10 mmol) and sodium-(L)-ascorbate (0.21 mmol) were added subsequently to the solution. The reaction mixture was stirred at 50 °C for 18 hours. Solvents were removed under reduced pressure and the crude was purified through flash column chromatography





5-(2-Oxo-hexahydro-thieno[3,4-d]imidazol-4-yl)-pentanoic acid {3-[4-(3-{4-[3-(2,4-bis-allyloxy-phenyl)-5,6-dihydro-4H-pyridazine-1-sulfonyl]-phenylcarbamoyl}-propyl)-[1,2,3]triazol-1-yl]-propyl}-amide (14): The product was purified through flash column chromatography (from 0% to 5% methanol in DCM) to afford a colourless foam (69 mg, 0.081 mmol, 90%). ^1^H-NMR (MeOD 400 MHz) δ 7.80 (5H, m), 7.10 (1H, d, J = 8.2 Hz), 6.54 - 6.51 (2H, m), 6.06 (1H, ddt, J = 17.3 Hz, 10.5 Hz, 5.2 Hz), 5.98 (1H, ddt, J = 17.2 Hz, 10.4 Hz, 5.1 Hz), 5.40 (1H, dq, J = 17.2 Hz, 1.5 Hz), 5.30 (1H, dq, J = 17.2 Hz, 1.6 Hz), 5.27 (1H, dq, J = 10.5 Hz, 1.5 Hz), 5.21 (1H, dq, J = 10.6 Hz, 1.6 Hz), 4.55 (2H, dt, J = 5.2 Hz, 1.6 Hz), 4.50 (2H, dt, J = 5.0 Hz, 1.5 Hz), 4.48 (1H, dd, J = 7.8 Hz, 4.2 Hz), 4.40 (2H, t, J = 6.8 Hz), 4.29 (1H, dd, J = 7.7 Hz, 4.3 Hz), 3.38 (2H, t, J = 5.6 Hz), 3.21 - 3.16 (3H, m), 2.91 (1H, dd, J = 12.7 Hz, 4.9 Hz), 2.80 (2H, t, J = 7.3 Hz), 2.70 (1H, d, J = 12.7 Hz), 2.54 (2H, t, J = 6.6 Hz), 2.47 (2H, t, J = 7.3 Hz), 2.21 (2H, t, J = 7.3 Hz), 2.11 - 1.98 (4H, m), 1.75 - 1.55 (6H, m), 1.44 (2H, quint., J = 7.5 Hz). ^13^C-NMR (MeOD 126 MHz) δ 174.7, 172.8, 164.6, 160.6, 157.4, 155.1, 143.4, 133.3, 133.0, 129.9, 129.7, 129.2, 128.9, 122.2, 120.9, 118.8, 116.3, 116.2, 106.0, 100.2, 68.9, 68.5, 61.9, 60.2, 55.5, 43.2, 39.6, 35.9, 35.7, 35.3, 29.6, 28.3, 28.0, 25.8, 25.3, 24.8, 24.2, 18.6. IR (neat) ν_max_: 3311, 2391, 2060, 1684, 1162 cm^-1^. HRMS (ESI) calcd for C_41_H_53_N_9_NaO_7_S_2_ [M+Na]^+^ 870.3402 m/z found 870.3380 m/z.





5-(2-Oxo-hexahydro-thieno[3,4-d]imidazol-4-yl)-pentanoic acid {3-[4-(3-{4-[4-(2,4-bis-allyloxy-phenyl)-1H-phthalazine-2-sulfonyl]-phenylcarbamoyl}-propyl)-[1,2,3]triazol-1-yl]-propyl}-amide (15): The product was purified through flash column chromatography (from 0% to 5% methanol in DCM) to afford a colourless foam (40 mg, 0.044 mmol, 45%). ^1^H-NMR (Acetone 400 MHz) δ 9.95 (1H, s, CONH), 7.92 (2H, d, J = 9.0 Hz), 7.88 (2H, d, J = 8.9 Hz), 7.80 (1H, s), 7.49 (1H, t, J = 7.3 Hz), 7.44 (1H, d, J = 7.1 Hz), 7.39 (1H, d, J = 8.2 Hz), 7.32 (1H, t, J = 7.5 Hz), 7.00 (1H, d, J = 7.5 Hz), 6.73 (1H, dd, J = 8.2 Hz, 2.3 Hz), 6.67 (1H, d, J = 2.2 Hz), 6.14 (1H, ddt, J = 17.2 Hz, 10.5 Hz, 5.2 Hz), 5.99 (1H, s), 5.78 (1H, s), 5.63 (1H, ddt, J = 17.2 Hz, 10.8 Hz, 4.7 Hz), 5.48 (1H, dq, J = 17.2 Hz, 1.6 Hz), 5.31 (1H, dq, J = 10.5 Hz, 1.6 Hz), 4.88 (1H, dq, J = 10.6 Hz, 1.7 Hz), 4.82 (1H, dq, J = 17.3 Hz, 1.8 Hz), 4.68 (2H, dt, J = 5.4 Hz, 1.6 Hz), 4.54 - 4.50 (1H, m), 4.47 (2H, s), 4.44 - 4.39 (4H, m), 4.37 - 4.32 (1H, m), 3.23 - 3.15 (5H, m), 2.94 (1H, dd, J = 12.5 Hz, 4.9 Hz), 2.75 (2H, t, J = 7.1 Hz), 2.71 (1H, d, J = 12.5 Hz), 2.45 (2H, t, J = 7.3 Hz), 2.22 (2H, t, J = 7.0 Hz), 2.03 (2H, quint., J = 7.0 Hz), 1.86 - 1.40 (8H, m). ^13^C-NMR (Acetone 126 MHz) δ 172.8, 171.6, 164.0, 161.1, 157.8, 156.9, 146.4, 146.2, 144.4, 133.6, 132.8, 131.5, 131.2, 131.1, 129.9, 128.8, 128.0, 126.5, 126.0, 125.5, 121.9, 118.5, 118.4, 116.7, 115.6, 106.5, 100.4, 68.6, 68.4, 61.4, 60.0, 55.4, 47.1, 45.9, 40.1, 35.8, 35.7, 35.3, 30.1, 28.0, 25.4, 24.8, 24.4. IR (neat) ν_max_: 3288, 2359, 1692, 1590, 1166 cm^-1^. HRMS (ESI) calcd for C_45_H_53_N_9_NaO_7_S_2_ [M+Na]^+^ 918.3433 m/z found 918.3476 m/z.

### **General procedure for deallylation:**

The desired labelled bis-allyloxy derivative (**14** or **15**) (0.07 mmol) was dissolved in dry methanol (5 mL) and a catalytic amount of tetrakis(triphenylposphine)palladium was added. The reaction mixture at 60 °C until total consumption of starting material (2-3 hours). After cooling to room temperature, the solvent was removed under reduced pressure and the product was purified through flash column chromatography





5-(2-Oxo-hexahydro-thieno[3,4-d]imidazol-4-yl)-pentanoic acid {3-[4-(3-{4-[3-(2,4-dihydroxy-phenyl)-5,6-dihydro-4H-pyridazine-1-sulfonyl]-phenylcarbamoyl}-propyl)-[1,2,3]triazol-1-yl]-propyl}-amide (Biotin-RCZ12): The product was purified through flash column chromatography (from 0% to 7% methanol in DCM) to afford a white paste (28 mg, 0.035 mmol, 42%). ^1^H-NMR (MeOD 400 MHz) δ 7.80 (5H, m), 7.24 (1H, d, J = 9.2 Hz), 6.32 - 6.30 (2H, m), 4.61 (1H, s), 4.48 (1H, dd, J = 7.6 Hz, 4.4 Hz), 4.38 (2H, t, J = 6.8 Hz), 4.28 (1H, dd, J = 7.8 Hz, 4.4 Hz), 3.38 (2H, t, J = 5.4 Hz), 3.20 - 3.17 (3H, m), 2.90 (1H, dd, J = 12.8 Hz, 5.0 Hz), 2.78 (2H, t, J = 7.4 Hz), 2.69 (2H, d, J = 12.9 Hz), 2.60 (2H, t, J = 6.6 Hz), 2.44 (2H, t, J = 7.4 Hz), 2.21 (2H, t, J = 7.4 Hz), 2.14 - 2.02 (4H, m), 1.76 - 1.55 (4H, m), 1.43 (2H, quint., J = 7.6 Hz). ^13^C-NMR (MeOD 126 MHz) δ 174.8, 174.0, 172.8, 160.1, 159.9, 155.4, 146.8, 143.7, 134.5, 128.9, 127.9, 125.8, 122.2, 119.1, 106.7, 102.9, 61.9, 60.2, 55.5, 49.8, 42.9, 39.6, 35.9, 35.5, 35.3, 29.5, 28.3, 28.0, 25.3, 24.7, 24.2, 21.5, 18.2. IR (neat) ν_max_: 3283, 2932, 2359, 1676, 1355 cm^-1^. HRMS (ESI) calcd for C_35_H_45_N_9_NaO_7_S_2_ [M+Na]^+^ 790.2776 m/z found 790.2737 m/z.





5-(2-Oxo-hexahydro-thieno[3,4-d]imidazol-4-yl)-pentanoic acid {3-[4-(3-{4-[4-(2,4-dihydroxy-phenyl)-1H-phthalazine-2-sulfonyl]-phenylcarbamoyl}-propyl)-[1,2,3]triazol-1-yl]-propyl}-amide (Biotin-RCZ20): The product was purified through flash column chromatography (from 0% to 7% methanol in DCM) to afford a white paste (8 mg, 0.009 mmol, 47%). ^1^H-NMR (MeOD 400 MHz) δ 7.91 (2H, d, J = 9.1 Hz), 7.83 (2H, d, J = 8.9 Hz), 7.79 (1H, s), 7.57 - 7.54 (1H, m), 7.45 - 7.41 (3H, m), 7.26 (1H, d, J = 8.6 Hz), 6.44 (1H, d, J = 2.3 Hz), 6.39 (1H, dd, J = 8.5 Hz, 2.3 Hz), 4.47 (1H, dd, J = 7.8 Hz, 4.2 Hz), 4.37 (2H, t, J = 7.0 Hz), 4.31 (2H, s), 4.28 (1H, dd, J = 8.0 Hz, 4.4 Hz), 3.20 - 3.15 (3H, m), 2.90 (1H, dd, J = 12.6 Hz, 4.8 Hz), 2.78 (2H, t, J = 7.4 Hz), 2.68 (1H, d, J = 12.6 Hz), 2.45 (2H, t, J = 7.4 Hz), 2.20 (2H, t, J = 7.4 Hz), 2.04 (2H, quint., J = 6.9 Hz), 1.75 - 1.55 (6H, m), 1.43 (2H, quint., J = 7.1 Hz). ^13^C-NMR (MeOD 126 MHz) δ 172.7, 171.6, 163.3, 160.3, 157.8, 155.7, 147.4, 146.2, 144.9, 133.5, 132.1, 131.8, 129.8, 128.2, 127.4, 126.5, 125.8, 124.7, 122.0, 118.4, 106.9, 103.7, 61.3, 60.3, 55.4, 47.0, 46.4, 40.0, 35.8, 35.6, 35.3, 30.0, 29.2, 25.4, 24.7, 24.4. IR (neat) ν_max_: 3263, 2929, 2361, 1629, 1166 cm^-1^. HRMS (ESI) calcd for C_39_H_45_N_9_NaO_7_S_2_ [M+Na]^+^ 838.2891 m/z found 838.2911 m/z.

### **Table S1:** List of strains generated and used in this study.

| **Strain** | **Description and genotype** | **Source** |
| --- | --- | --- |
| TUV 93-0 | Derivative of EHEC O157:H7 strain EDL933 Shiga toxin negative (stx) | (Campellone, Robbins, & Leong, 2004) |
| TUV 93-0 EspD-HA | Derivative of TUV93-0 containing chromosomal EspD-HA | This study |
| TUV 93-0 EspD-HA_Δ26-81_ | Derivative of TUV93-0 containing chromosomal EspDHA_Δ26-81_ (Amphipathic domains 1 & 2 deleted) | This study |
| TUV 93-0 EspD-HA_Δ138-171_ | Derivative of TUV93-0 containing chromosomal EspDHA_Δ138-171_ (Coiled-coil 1 domain deleted) | This study |
| TUV 93-0 EspD-HA_Δ176-251_ | Derivative of TUV93-0 containing chromosomal EspDHA_Δ176-251_ (Transmembrane domains 1 & 2 deleted) | This study |
| TUV 93-0 EspD-HA_Δ333-351_ | Derivative of TUV93-0 containing chromosomal EspDHA_Δ333-356_ (Coiled-coil 2 domain deleted) | This study |
| Strataclone® Competent Cells | DH5-α Competent cells Tetr *Δ*(*mcrA*)183 *Δ*(*mcrCB-hsdSMR-mrr*)173 *endA1 supE44 thi-1 recA1 gyrA96 relA1 lac Hte* [F’ *proAB* laclqZ*ΔM15* Tn*10* (Tet^r^) Amy Cam^r^] | Agilent |
| One Shot® TOP10 Competent Cells | Chemically competent cells F^-^ *mcrA* Δ(*mrr-hsd*RMS-*mcr*BC) F80*lac*ZΔM15 Δ*lac*X74 *recA1 ara*Δ139 Δ(*ara-leu*)7697 *gal*U *gal*K *rps*L (Str^R^) *endA1 nup*G | Invitrogen |
| NEB Competent Cells | DH5- α Competent cells *fhuA2* Δ*(argF-lacZ)U169 phoA glnV44 Φ80* Δ*(lacZ)M15 gyrA96 recA1 relA1 endA1 thi-1 hsdR17* | New England Biolabs |

### **Table S2:** List of plasmids generated and used in this study.

| **Plasmid** | **Description** | **Selection** | **Source** |
| --- | --- | --- | --- |
| pIB307 | Allelic exchange vector; temperature sensitive | Chl | (Emmerson et al., 2006) |
| pIB307-*SacBKan* | Recombinant temperature sensitive plasmid bearing *SacBKan* cassette | Chl/Kan | (Emmerson et al., 2006) |
| pIB307-*LEE4*-EspD-HA | pIB307 cut with *Sac*1/*Pst*1, *LEE4-espDHA* construct cloned into *Sac*1/*Pst*1 | Chl | This study |
| pIB307-*LEE4*-*SacBKan* | pIB307-*LEE4-*EspDHA cut with *BamH*1, *SacKan* gene cloned into the *BamH*1 span | Chl/Kan | This study |
| pIB307-*LEE4*-EspD-HA_Δ26-81_ | pIB307-*LEE4*-*SacBKan* cut with *BamH*1/*ApaL*1, *espDHA*Frg1 and *espDHA*Frg2 fragments assembled into *BamH*1/*ApaL*1 | Chl | This study |
| pIB307-*LEE4*- EspD-HA_Δ138-171_ | pIB307-*LEE4*- *SacBKan* cut with *BamH*1/*ApaL*1, *espDHA*Frg3 and *espDHA*Frg4 fragments assembled into *BamH*1/*ApaL*1 | Chl | This study |
| pIB307-*LEE4*- EspD-HA_Δ176-251_ | pIB307-*LEE4*- *SacBKan* cut with *BamH*1/*ApaL*1, *espDHA*Frg5 and *espDHA*Frg6 fragments assembled into *BamH*1/*ApaL*1 | Chl | This study |
| pIB307-*LEE4*- EspD-HA_Δ333-356_ | pIB307-*LEE4*- *SacBKan* cut with *BamH*1/*ApaL*1, *espDHA*Frg7 and *espDHA*Frg8 fragments assembled into *BamH*1/*ApaL*1 | Chl | This study |

### **Table S3:** List of oligonucleotides used in this study.

| **Primer name** | **Primer sequence** |
| --- | --- |
| pIB307-*LEE4*-EspD-HA1 F | CGGACTGTTGTAACTCAG |
| pIB307-*LEE4*-EspD-HA1 R/ pIB307-*LEE4*-EspD-HA2 R | TGCGACTCCTGCATTAGG |
| pIB307-*LEE4*-EspD-HA2 F | CCGTGACCAGTCTTGCTG |
| *BamH1* Span F | CCATCGTTACTTGAGTATTATC |
| *BamH1* Span R | TTCAGCAAATGTCGAATAC |
| *espD-HA*Frg1 F | GTGCGACTAGATTGAGTAATACCAGAAGTACC |
| *espD-HA*Frg1 R | CATTAAATCTCTTAACGAGGTGCACGTTCTGATGTGC |
| *espD-HA*Frg2 F | AATAAAATTCTCTTTAATAAGGGATCCTTAAGCGTAATCTGGAAC |
| *espD-HA*Frg2 R | ACTCAATCTAGTCGCACTGAGGAGGCA |
| *espD-HA*Frg3 F | TGGCCAACCATATAAATCTCGTTAGAGTTTTTCAG |
| *espD-HA*Frg3 R | CATTAAATCTCTTAACGAGGTGCACGTTCTGATGTGC |
| *espD-HA*Frg4 F | AATAAAATTCTCTTTAATAAGGGATCCTTAAGCGTAATCTGG |
| *espD-HA*Frg4 R | GATTTATATGGTTGGCCAGGTCTTTGGT |
| *espD-HA*Frg5 F | TACCAAATTTAAAGACCTGGCCAACAATTTTAC |
| *espD-HA*Frg5 R | CATTAAATCTCTTAACGAGGTGCACGTTCTGATGTGC |
| *espD-HA*Frg6 F | AATAAAATTCTCTTTAATAAGGGATCCTTAAGCGTAATCTG |
| *espD-HA*Frg6 R | CAGGTCTTTAAATTTGGTAATGTTGCTAACAAAATTG |
| *espD-HA*Frg7 F | CATCAGTTGACTCTCACCTAAGCTTTCAC |
| *espD-HA*Frg7 R | CATTAAATCTCTTAACGAGGTGCACGTTCTGATGTGC |
| *espD-HA*Frg8 F | AATAAAATTCTCTTTAATAAGGGATCCTTAAGCGTAATCTG |
| *espD-HA*Frg8 R | GTGAGAGTCAACTGATGTCCGATTCAG |

**Table S4.** Summary of significantly (p < 0.05) differentially expression genes identified by RNA-seq

| **Feature ID** | **Gene description** | **Biological function** | **Fold change** | **FDR p-value** | **Mean expression WT*** | **Mean expression RCZ20**** |
| --- | --- | --- | --- | --- | --- | --- |
| *torC* | Cytochrome c-like | Iron acquisition | 15.97 | 5.74E-10 | 1 | 18 |
| *torA* | Molybdopterin guanine dinucleotide-containing S/N-oxide reductase | Redox processes | 13.44 | 1.63E-31 | 4.67 | 64.67 |
| *yjiY* | Carbon starvation protein | Stress responses | 6.64 | 1.88E-16 | 9.67 | 65.67 |
| *metF* | Methylenetetrahydrafolate reductase | Amino acid metabolism | 4.83 | 2.63E-09 | 6 | 29.67 |
| *bssR* | Biofilm regulator | Transcriptional regulation | 4.51 | 1.73E-06 | 4.67 | 21.67 |
| *Z2427* | Unknown | Hypothetical | 2.64 | 3.63E-16 | 115 | 308.33 |
| *metE* | Homocysteien methyltransferase | Amino acid metabolism | 2.61 | 5.75E-16 | 254 | 658.33 |
| *yhiW* | Regulator of the GAD response | Transcriptional regulation | 2.48 | 8.34E-04 | 15.33 | 38.33 |
| *ydaJ* | Aminohydrolase | General metabolism | 2.38 | 1.47E-15 | 196 | 468.67 |
| *yhfC* | MFS transporter | Membrane transport | 2.37 | 2.71E-07 | 25.33 | 60.67 |
| *Z2428* | Unknown | Hypothetical | 2.12 | 2.12E-07 | 45.33 | 97.33 |
| *ycfS* | L/D transpeptidase | Membrane associated | 1.89 | 0.02 | 20.33 | 39 |
| *htrL* | LPS biosynthetic enzyme | LPS metabolism | 1.79 | 0.04 | 20 | 36.33 |
| *metK* | S-adenosylmethionine synthase | Amino acid metabolism | 1.75 | 3.46E-05 | 130 | 229.67 |
| *ydeA* | Putative sugar efflux | Membrane transport | 1.75 | 0.05 | 22.33 | 39.33 |
| *yjbA* | Phosphate starvation protein | Stress responses | 1.75 | 6.50E-03 | 34 | 59.67 |
| *gcd* | Glucose dehydrogenase | Redox processes | 1.72 | 2.22E-04 | 101.33 | 174.33 |
| *metN* | Methionine import ATP-binding | Membrane transport | 1.71 | 0.04 | 22.33 | 38.67 |
| *yihE* | Stress response kinase | Phosphorylation | 1.66 | 1.03E-03 | 51.67 | 86.33 |
| *yojH* | Malate oxidoreductase | TCA cycle | 1.64 | 5.21E-04 | 121.67 | 200.33 |
| *cpxP* | CPX signalling chaperone | Signal transduction | 1.62 | 1.03E-03 | 101.67 | 166.67 |
| *dps* | DNA protection | Stress responses | 1.6 | 2.22E-04 | 383.33 | 614.67 |
| *yfiA* | Ribosome hibernation protein | General metabolism | 1.57 | 4.66E-04 | 308.67 | 492.33 |
| *folE* | GTP cyclohydrolase | Carbohydrate metabolism | 1.57 | 6.96E-03 | 101.33 | 161 |
| *ompC* | Outer membrane protein | Membrane transport | 1.52 | 0.02 | 2,601.67 | 3,892.33 |
| *ompX* | Outer membrane protein | Membrane associated | 1.52 | 8.64E-04 | 304.67 | 465 |
| *Z2779* | Unknown | Hypothetical | 1.38 | 0.02 | 489.33 | 677.67 |
| *ydjS* | Succinylglutamate desuccinylase | Amino acid metabolism | 1.38 | 0.02 | 361.67 | 501.67 |
| *Z2777* | Unknown | Hypothetical | 1.37 | 0.02 | 692.33 | 953 |
| *astD* | Succinylglutamate dehydrogenase | Amino acid metabolism | 1.37 | 0.02 | 781 | 1,072.67 |
| *argD_1* | Transaminase | Amino acid metabolism | 1.35 | 0.02 | 748.33 | 1,022.00 |
| *ptsH* | Phosphotransferase system | Phosphorylation | -1.4 | 0.02 | 467.33 | 336 |
| *Z1824* | Unknown | Hypothetical | -1.4 | 0.02 | 378.33 | 271 |
| *escN* | Locus of enterocyte effacement island | LEE-associated | -1.5 | 5.59E-03 | 261.33 | 177 |
| *Orf4* | Locus of enterocyte effacement island | LEE-associated | -1.53 | 0.04 | 168.33 | 113.67 |
| *SepD* | Locus of enterocyte effacement island | LEE-associated | -1.53 | 0.02 | 111 | 73.67 |
| *Z6020* | Unknown | Hypothetical | -1.54 | 0.04 | 130.33 | 86.67 |
| *Map* | Locus of enterocyte effacement island | LEE-associated | -1.54 | 7.79E-03 | 207.67 | 138 |
| *cesD2* | Locus of enterocyte effacement island | LEE-associated | -1.55 | 4.62E-03 | 1,151.67 | 766.33 |
| *espD* | Locus of enterocyte effacement island | LEE-associated | -1.55 | 0.01 | 5,349.33 | 3,568.00 |
| *espA* | Locus of enterocyte effacement island | LEE-associated | -1.57 | 8.63E-03 | 3,605.00 | 2,373.33 |
| *espH* | Locus of enterocyte effacement island | LEE-associated | -1.59 | 6.45E-03 | 215.33 | 139.67 |
| *Z3920* | Unknown | Hypothetical | -1.59 | 1.03E-03 | 186.33 | 118.67 |
| *cesT* | Locus of enterocyte effacement island | LEE-associated | -1.6 | 5.91E-04 | 760.67 | 484 |
| *Z5352* | Unknown | Hypothetical | -1.6 | 0.01 | 74.67 | 47 |
| *Z3644* | Unknown | Hypothetical | -1.6 | 6.50E-03 | 450 | 276.67 |
| *eae* | Locus of enterocyte effacement island | LEE-associated | -1.61 | 5.13E-05 | 5,977.33 | 3,781.67 |
| *tir* | Locus of enterocyte effacement island | LEE-associated | -1.62 | 9.84E-04 | 2,347.67 | 1,491.33 |
| *ptsG* | Phosphotransferase system | Phosphorylation | -1.63 | 3.34E-05 | 453.33 | 282.33 |
| *espF* | Locus of enterocyte effacement island | LEE-associated | -1.63 | 3.29E-03 | 395.33 | 249 |
| *espB* | Locus of enterocyte effacement island | LEE-associated | -1.64 | 3.61E-03 | 5,494.00 | 3,461.67 |
| *escC* | Locus of enterocyte effacement island | LEE-associated | -1.64 | 1.03E-03 | 330 | 205.33 |
| *escI* | Locus of enterocyte effacement island | LEE-associated | -1.65 | 4.06E-03 | 159.33 | 98.67 |
| *Z3071* | Unknown | Hypothetical | -1.66 | 2.71E-03 | 110.67 | 67.33 |
| *escF* | Locus of enterocyte effacement island | LEE-associated | -1.66 | 0.02 | 164 | 102.67 |
| *sepL* | Locus of enterocyte effacement island | LEE-associated | -1.66 | 6.47E-05 | 558.33 | 344.67 |
| *ler* | Locus of enterocyte effacement island | LEE-associated | -1.69 | 1.78E-03 | 144.67 | 87.33 |
| *manZ* | Phosphotransferase system | Membrane transport | -1.71 | 3.11E-06 | 415.33 | 245 |
| *cesA2* | Locus of enterocyte effacement island | LEE-associated | -1.72 | 4.04E-03 | 155 | 92.67 |
| *manY* | Phosphotransferase system | Membrane transport | -1.75 | 4.39E-06 | 354.33 | 204.67 |
| *grlA* | Locus of enterocyte effacement island | LEE-associated | -1.75 | 2.34E-03 | 203.67 | 120.33 |
| *sepQ* | Locus of enterocyte effacement island | LEE-associated | -1.75 | 7.81E-04 | 225.33 | 132 |
| *Z0955* | Unknown | Hypothetical | -1.76 | 9.23E-05 | 493 | 287.67 |
| *Z4326* | Unknown | Hypothetical | -1.79 | 0.02 | 57.67 | 33 |
| *manX* | Phosphotransferase system | Membrane transport | -1.88 | 6.86E-09 | 390.67 | 211.33 |
| *espG* | Locus of enterocyte effacement island | LEE-associated | -1.9 | 1.03E-03 | 187.67 | 103 |
| *rcsA* | RCS phosphorelay system regulator | Transcriptional regulation | -1.94 | 2.54E-05 | 75 | 39 |
| *cesD* | Locus of enterocyte effacement island | LEE-associated | -1.95 | 3.34E-05 | 117.67 | 61.33 |
| *escR* | Locus of enterocyte effacement island | LEE-associated | -1.95 | 4.62E-03 | 60 | 31.67 |
| *cesAB* | Locus of enterocyte effacement island | LEE-associated | -1.97 | 1.21E-03 | 71 | 36.67 |
| *gcvH* | Glycine cleavage system protein | General metabolism | -2 | 0.04 | 28.67 | 14.33 |
| *escL* | Locus of enterocyte effacement island | LEE-associated | -2.04 | 1.89E-07 | 98.33 | 48.67 |
| *orf1* | Locus of enterocyte effacement island | LEE-associated | -2.31 | 0.02 | 32 | 14.33 |
| *wcaI* | Colanic acid biosynthetic protein | General metabolism | -2.4 | 0.02 | 24 | 10 |
| *escE* | Locus of enterocyte effacement island | LEE-associated | -2.42 | 7.24E-07 | 59 | 24.67 |
| *wcaD* | Colanic acid biosynthetic protein | Membrane associated | -2.47 | 0.02 | 17.33 | 7 |
| *fruA* | Fructose transporter subunit | Membrane transport | -2.49 | 2.78E-07 | 62.67 | 25.33 |
| *cpsB* | Mannose-1-phosphate guanyltransferase | General metabolism | -2.54 | 6.96E-03 | 20.33 | 8 |
| *wcaG* | L-fucose synthase | Carbohydrate metabolism | -2.67 | 6.47E-05 | 32.67 | 12.33 |
| *Z3921* | Unknown | Hypothetical | -2.74 | 2.17E-03 | 21 | 7.67 |
| *Z2151* | Unknown | Hypothetical | -2.79 | 3.49E-03 | 21.33 | 7.67 |
| *fruK* | Phosphofructokinase | Carbohydrate metabolism | -3.15 | 3.30E-04 | 35 | 11.33 |
| *Z2339* | Unknown | Hypothetical | -3.16 | 1.51E-03 | 18 | 5.67 |
| *gmd* | Mannose dehydratase | General metabolism | -3.39 | 2.81E-10 | 55 | 16.33 |
| *Z6024* | Unknown | Hypothetical | -3.52 | 1.03E-11 | 109.67 | 32.33 |
| *fruB* | Phosphotransferase system | Carbohydrate metabolism | -3.75 | 1.79E-08 | 35 | 9.33 |
| *wcaH* | Mannose hydrolase | Carbohydrate metabolism | -4.26 | 4.10E-03 | 11.67 | 2.67 |

* = WT EHEC cultured in MEM-HEPES to mid exponential phase (n = 3)

** = WT EHEC cultured in MEM-HEPES + 50 μM RCZ20 to mid exponential phase (n = 3)

A

B

**Fig S1.** (A) Summarised synthetic map of the RCZ compound derivatives and intermediates. Compounds labelled in blue and their associated modifications are indicated on the schematic in blue. (B) The miLogP values for RCZ12, RCZ20 and ME055 are also indicated.


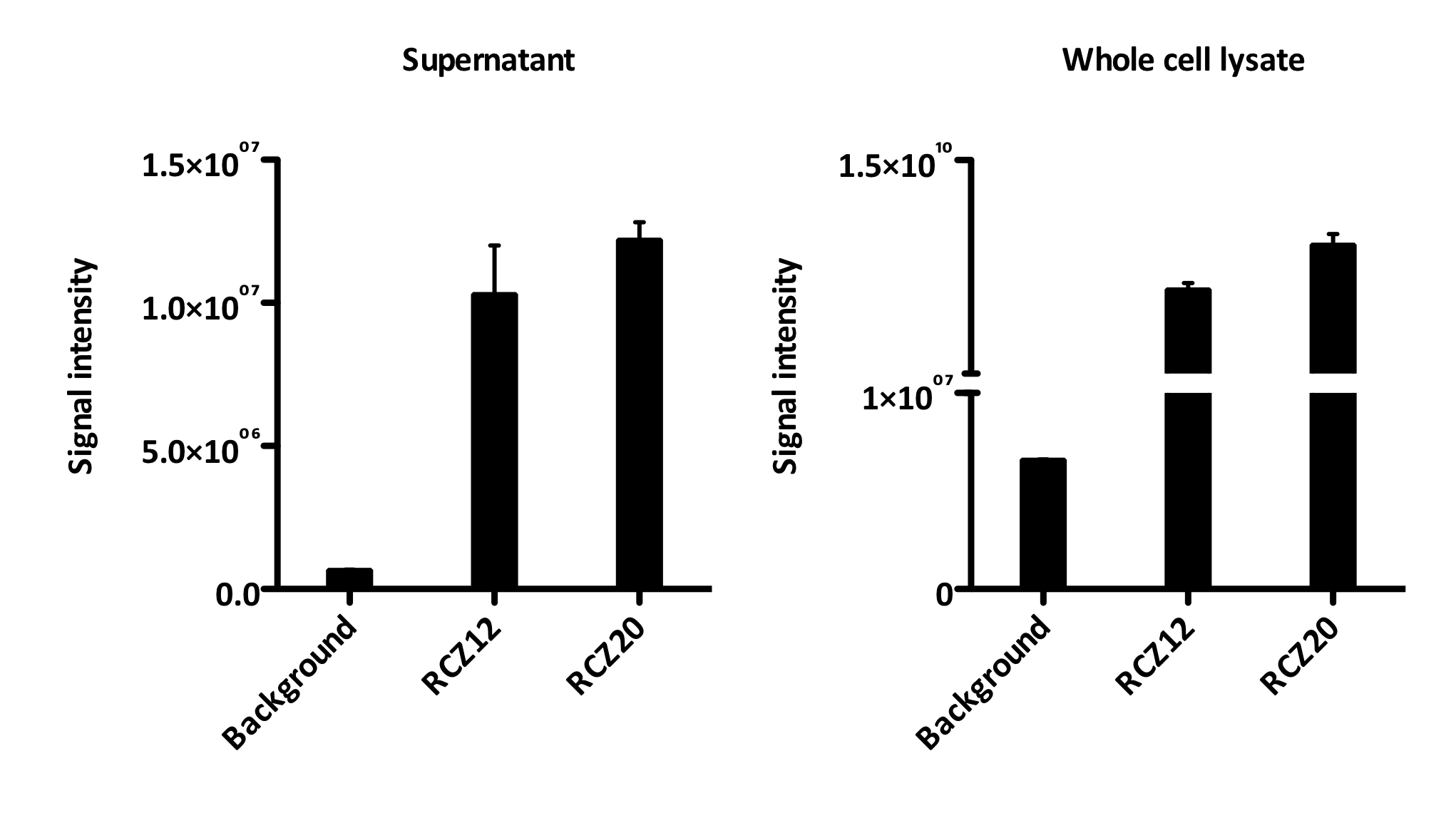


**Fig S2**. Targeted metabolomic profiling data for RCZ12 and RCZ20 cellular uptake. Average intensities of RCZ12 and RCZ20 (± corresponding standard deviation) in supernatant and whole cell lysates. Untreated bacterial cell cultures were included as a background control for signal noise. Experiments were performed in biological triplicate and analysed through hydrophilic interaction liquid chromatography (HILIC).

A

B

C

D

E

**Fig S3**. Stability of ME055 and RCZ12 after exposure to acidic conditions. 1H NMR spectra of ME0055 in DMSO alone (A) , ME0055 in DMSO + acid after 2 h incubation (B), ME0055 in DMSO + acid after 12 hours incubation (C), RCZ12 in DMSO (D) and RCZ in DMSO + acid after 12 hours incubation (E).

**Fig S4.** Summarised synthetic map of biotin labelled RCZ12 and RCZ20 compound derivatives and intermediates. Compounds labelled in blue and their associated modifications are indicated on the schematic in blue.
